# Injectable SF-platform orchestrates GPX4-targeted ferroptosis-autophagy-immunogenic circuit for overcoming oxidative resistance in triple-negative breast cancer

**DOI:** 10.7150/thno.116013

**Published:** 2025-08-11

**Authors:** Hui Yuan, Xiongwu Li, Muhua Yu, Youde Cao, Lingcheng Wu, Suyujie Shi, Yaying Yang, Kexiao Yu, Bing Liang

**Affiliations:** 1 Department of Pathology from the College of Basic Medicine, and Clinical Pathology Laboratory of Pathology Diagnostic Center, and Molecular Medicine Diagnostic & Testing Center, Chongqing Medical University, 1 Yixueyuan Road, Yuzhong Distinct, Chongqing 400016, China.; 2 Department of Pathology, the First Affiliated Hospital of Chongqing Medical University, 1 Youyi Road, Yuzhong Distinct, Chongqing, 400042, China.; 3 Department of Pathology, The People's Hospital of Dazu, Chongqing; The Affiliated Dazu's Hospital of Chongqing Medical University, Chongqing, 402360, China.; 4 Department of Obstetrics and Gynecology, The Second Affiliated Hospital of Chongqing Medical University, Chongqing, 400010, China.; 5 Department of Orthopedics, Chongqing Traditional Chinese Medicine Hospital, the First Affiliated Hospital of Chongqing College of Traditional Chinese Medicine, No. 6 Panxi Seventh Branch Road, Jiangbei District, Chongqing 400021, China.

**Keywords:** Injectable SF-Platform, ROS, Ferroptosis, Autophagy, ICD

## Abstract

**Background:** Triple-negative breast cancer (TNBC) is the most aggressive subtype of breast cancer, characterized by the absence of targeted therapies and poor clinical outcomes. The development of novel, effective therapeutic strategies is urgently required to improve patient prognosis. Ferroptosis, a regulated form of cell death, has recently emerged as a promising therapeutic approach for TNBC.

**Objectives:** This study aims to evaluate the efficacy of an injectable silk fibroin (SF)-based platform in treating TNBC and to explore the underlying mechanisms involved.

**Methods:** We engineered an injectable SF platform consisting of magnetic, thermoresponsive silk fibroin-based hydrogels (IMSFs) coupled with temporally controlled TAT-Beclin1 delivery. Ferroptosis induction was quantified using the C11-BODIPY probe and transmission electron microscopy (TEM). To investigate the molecular mechanisms, RNA sequencing, Western blotting, and co-immunoprecipitation were performed. Additionally, enzyme-linked immunosorbent assay (ELISA) and flow cytometry were used to assess immune responses.

**Results:** In the presence of an alternating magnetic field (AMF), IMSFs induce localized hyperthermia (42-45 °C) and catalyze Fenton reaction-driven reactive oxygen species (ROS) generation through Fe²⁺/Fe³⁺ release. The resulting ROS synergize with sorafenib release to inhibit the Xc⁻ system via the AMPK-Beclin1-SLC7A11 axis, leading to the local suppression of glutathione peroxidase 4 (GPX4) activity and the initiation of a ferroptosis cascade. Temporal delivery of TAT-Beclin1 prevents premature activation of autophagy, which could otherwise dampen ferroptosis. Instead, this approach leverages autophagy-ferroptosis synergy to amplify immunogenic cell death (ICD). This, in turn, activates CD8^+^ T cells, triggering interferon-gamma (IFN-γ)-mediated downregulation of SLC7A11 and establishing a self-amplifying cascade of “ferroptosis-autophagy-immunity” loop that induces oxidative stress and leads to enhanced anti-tumor effects.

**Conclusion:** The injectable SF platform, incorporating sequential therapeutic modules, demonstrates potent anti-tumor efficacy and holds significant promise for clinical translation in the treatment of TNBC.

## Introduction

Triple-negative breast cancer (TNBC) is the most aggressive subtype of breast cancer due to its absence of specific therapeutic targets, limited responsiveness to chemotherapy, and low immunogenicity. These characteristics significantly contribute to TNBC's propensity for recurrence and metastasis, posing considerable challenges for effective clinical management 1, 2.

Ferroptosis, a recently identified form of regulated cell death, has shown promising potential as an anti-cancer strategy 3, 4. This process involves iron-dependent lipid peroxidation (LPO) accumulation to cytotoxic levels 5, presenting a suitable therapeutic avenue for TNBC, which inherently exhibits elevated iron and lipid concentrations 6-8. During ferroptosis, polyunsaturated fatty acids (PUFAs) undergo extensive peroxidation, resulting in membrane lipid bilayer disruption and compromised cellular integrity 9, 10. However, glutathione peroxidase 4 (GPX4) inhibits ferroptosis by converting lipid peroxides into non-toxic lipid alcohols, thereby maintaining cellular homeostasis 11. Consequently, targeting GPX4 has emerged as an effective approach for ferroptosis induction. Nonetheless, potent and selective GPX4 inhibitors suitable for *in vivo* application remain rare, with none advancing to clinical use 12. GPX4 activity depends critically on intracellular glutathione (GSH) availability, synthesized via the system Xc⁻ transporter composed of the SLC7A11 and SLC3A2 subunits, which mediate cystine uptake 13. Thus, inhibiting system Xc⁻ to reduce intracellular GSH 14 represents a promising strategy to suppress GPX4.

Previous studies identified small molecules capable of inhibiting system Xc⁻, notably erastin and sorafenib (SOR) 15. Although erastin is commonly utilized experimentally, its poor *in vivo* compatibility and lack of clinical approval limit its translational potential 16. In contrast, sorafenib, approved as a first-line treatment for hepatocellular carcinoma (HCC), exerts potent ferroptosis-inducing activity. However, sorafenib's systemic distribution due to poor specificity results in significant adverse effects such as gastrointestinal discomfort, diarrhea, and hypertension in approximately 30% of patients 17. Additionally, its rapid metabolic clearance and low oral bioavailability (~8.43%) further restrict therapeutic efficacy 18. These limitations underscore the critical need for targeted and responsive drug-delivery platforms capable of achieving localized GPX4 suppression while minimizing systemic toxicity. Nanoparticle-based carriers, such as nanoparticles and hydrogels, have been extensively explored for drug delivery applications 19. Although nanoparticle-based systems can achieve stimulus-responsive drug release within tumor microenvironments, their efficacy largely depends on the enhanced permeability and retention (EPR) effect, particularly pronounced in TNBC. Nevertheless, nanoparticle efficacy is hampered by immune clearance, inconsistent EPR effects, and particle dispersion, severely limiting clinical translation 20-22. Conversely, implantable hydrogels circumvent systemic clearance but exhibit restricted drug-release capacities and substantial tissue damage upon implantation, hindering widespread clinical application. Injectable hyaluronic acid (HA)-based hydrogels possess excellent biocompatibility and safety profiles 23; however, rapid degradation and poor structural stability significantly limit their practical use 24, 25. Recently, composite hydrogels formed by crosslinking modified silk fibroin with HA have notably improved stability and even demonstrated electrically conductive properties for electrophysiological recordings 26. Despite these advancements, their complex fabrication and uncertain safety of crosslinking agents have impeded widespread adoption. Therefore, developing a novel, easy-to-prepare, safe, and reliable injectable composite hydrogel capable of localized, responsive drug release and inducing synergistic amplification of therapeutic effects and immune activation represents significant clinical potential.

This study introduces an innovative concept of a “local GPX4 suppressor” realized through the development of injectable magnetic thermoresponsive silk fibroin-based hydrogels (IMSFs). IMSFs are synthesized by co-encapsulating superparamagnetic nanoparticles (Fe₃O₄) and sorafenib within a silk fibroin-hyaluronic acid (SF-HA) hydrogel. The SF-HA composite forms a biomimetic extracellular matrix-like scaffold via protein-polysaccharide interactions 24, 25, enhancing structural stability and protecting encapsulated nanoparticles and drugs from shear forces during injection due to its porous, sponge-like architecture. Furthermore, unlike phototherapy or ultrasound, this platform enables precise control of magnetic hyperthermia temperature (MHT) generated by Fe₃O₄ nanoparticles at the tumor site through adjustments in alternating magnetic field (AMF) power. MHT provides targeted mild hyperthermia (42-45 °C), limiting thermal damage to surrounding healthy tissues 27. Importantly, AMF stimulation promotes iron ion (Fe²⁺/Fe³⁺) release from Fe₃O₄ nanoparticles within the acidic tumor microenvironment, facilitating intracellular iron accumulation and reactive oxygen species (ROS) generation via the Fenton reaction. Transcriptomic sequencing further demonstrated that sorafenib release inhibits the system Xc⁻ via the AMPK-Beclin1-SLC7A11 axis, achieving local GPX4 suppression. This integrated approach results in a synergistic ferroptosis cascade characterized by enhanced localized ROS production and disruption of the tumor's oxidative defense, while minimizing systemic toxicity.

Although ferroptosis is known to be immunogenic 3, its efficacy remains limited in low-immunogenic tumors such as TNBC, often termed “cold tumors” 28. Single-agent-induced ferroptosis typically fails to sufficiently trigger immunogenic cell death (ICD), compounded by potential premature drug depletion, contributing to TNBC's high recurrence and metastasis rates. Immune checkpoint inhibitors, such as PD-1 and PD-L1 antibodies, show promise in reshaping the tumor immune microenvironment 29; however, their clinical application is significantly hindered by high costs and severe immune-related adverse effects, including colitis and myasthenia gravis 30-32. Hence, strategies that markedly enhance ferroptosis efficacy to robustly activate ICD and achieve sustained anti-tumor responses are urgently required.

**Scheme 1:**
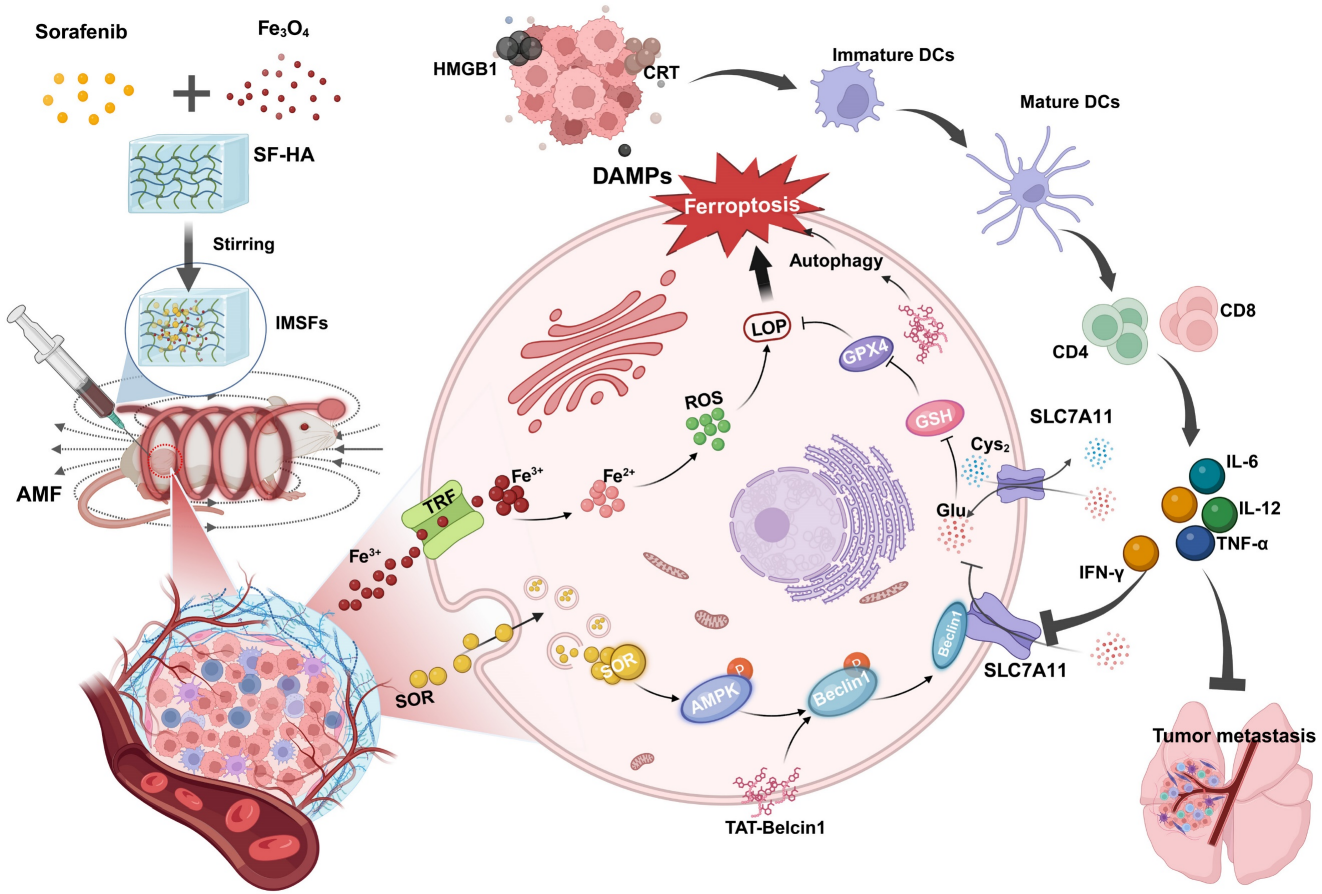
Illustration of injectable magnetic thermoresponsive silk fibroin-based hydrogels (IMSFs) combined with TAT-Beclin1 Injection enhancing ferroptosis and immune responses in TNBC cells. (This figure is created by biorender.)

Recent findings indicate that TAT-Beclin1, a membrane-permeable peptide, effectively induces autophagy via Beclin1 activation. Additionally, TAT-Beclin1 facilitates Beclin1-SLC7A11 complex formation 33, linking autophagy activation with ferroptosis enhancement. Based on these insights, we innovatively incorporated TAT-Beclin1 into our therapeutic regimen via intratumoral injection, employing a sequential treatment strategy: initial ferroptosis induction by IMSFs followed by autophagy activation via TAT-Beclin1(Scheme 1). This temporal control strategy ensures that autophagy does not prematurely neutralize ROS, which could otherwise inhibit ferroptosis progression 34-36. Instead, this sequential approach synergistically amplifies ferroptosis and autophagy, robustly triggering ICD. Subsequent ICD activation stimulates CD8⁺ T cells to produce IFN-γ, a cytokine that downregulates SLC7A11 expression, reinforcing ferroptosis induction 37. This establishes a self-amplifying “ferroptosis-autophagy-immunity-ferroptosis” cascade, orchestrated by the injectable SF-platform, effectively collapsing the oxidative defense of TNBC cells, thus achieving durable antitumor efficacy. Consequently, this dual-action therapy integrates localized ferroptosis with systemic immune activation, providing a novel therapeutic paradigm for aggressive TNBC and demonstrating strong translational potential for clinical applications.

## Results and Discussion

### Oral efficacy and toxicity evaluation of sorafenib

**Figure 1:**
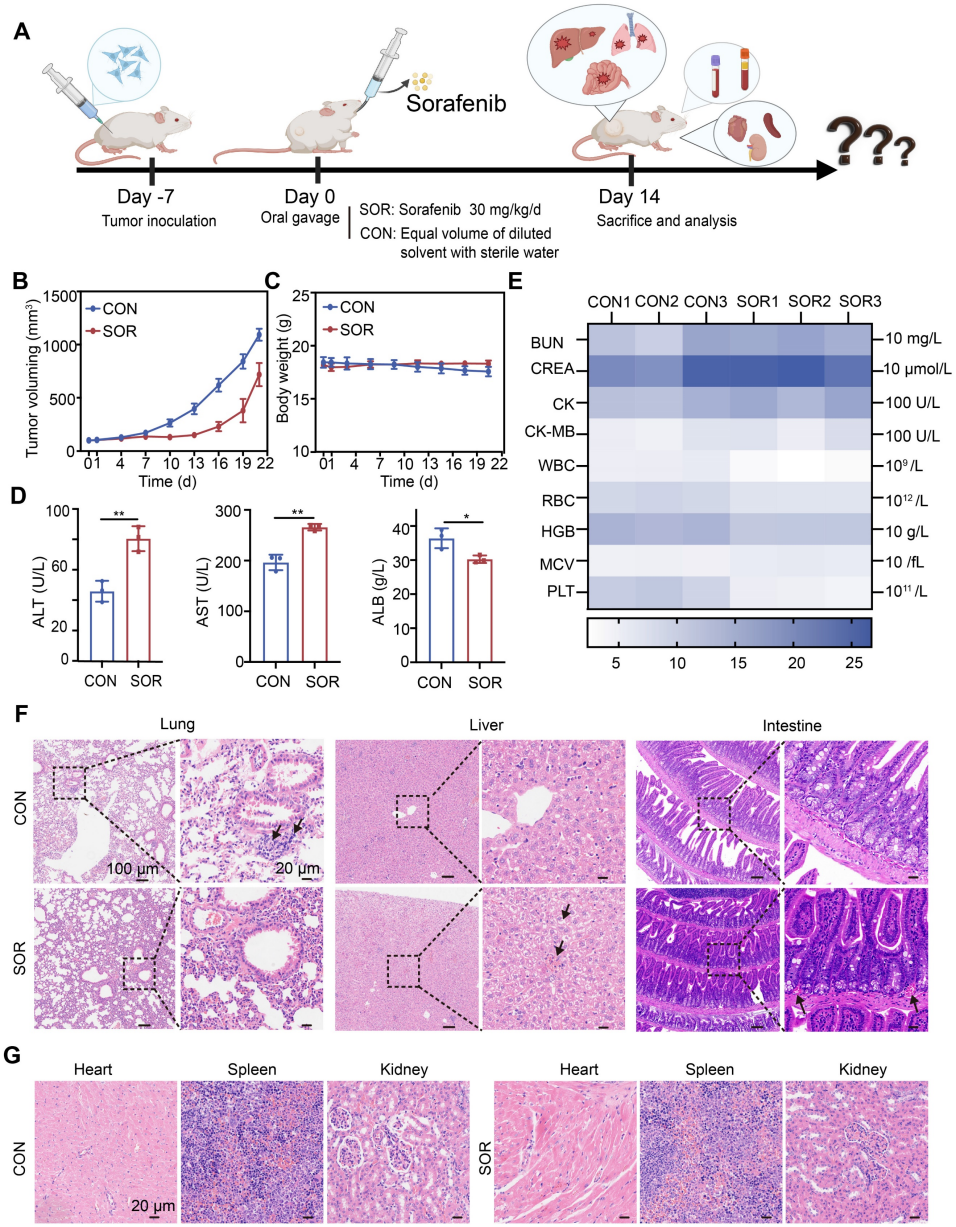
**Oral efficacy and toxicity study of sorafenib**. (A) Schematic illustration of the treatment process. (B) A curve representing tumor volume changes. (C) A curve representing body weight fluctuations. (D)ALT, AST, and ALB levels in serum, (E) Heat map of other blood routines, and blood biochemical indexes. (F, G) Representative H&E images of major organs (heart, liver, spleen, lung, and kidney) of BALB/c mice after different treatment groups. (The results are presented as mean values ± SDs, n = 3 per group. Statistical significance is indicated as follows: n.s. denotes non-significant differences, *p < 0.05, **p < 0.01 in the comparisons between groups.)

Ferroptosis induction is considered a promising therapeutic strategy for treating TNBC, an aggressive cancer subtype with limited treatment options. Here, we systematically evaluated the therapeutic efficacy and safety of oral sorafenib, a clinically approved ferroptosis inducer, using an immunocompetent orthotopic TNBC mouse model (BALB/c) (Figure 1A). Oral sorafenib (SOR group) exhibited moderate tumor growth inhibition compared to the control group (CON group), achieving less than 40% inhibition efficiency (Figure 1B and Figure S1). This modest efficacy likely results from poor oral bioavailability 38 and significant first-pass hepatic metabolism, common limitations of systemic administration. No statistically significant body weight differences were observed between groups (Figure 1C), suggesting systemic tolerability of the administered dose. Hematological and biochemical analyses revealed notable elevations in serum alanine aminotransferase (ALT) and aspartate aminotransferase (AST) levels, alongside reduced albumin (ALB) concentrations in the SOR group compared with controls (Figure 1D). Other blood parameters remained unaffected (Figure 1E). These observations indicate dose-dependent hepatotoxicity likely linked to sorafenib's hepatic metabolism post-oral administration.

Hematoxylin and eosin (H&E) staining of major organs demonstrated no lung metastasis in the SOR group; however, liver samples exhibited hepatic sinusoidal congestion and hepatocyte ballooning degeneration, accompanied by significant vascular congestion in the intestinal wall (Figure 1F). Other organs, including the heart, spleen, and kidneys, showed no significant pathological alterations (Figure 1G). Collectively, oral sorafenib demonstrated potential therapeutic activity against TNBC metastasis but induced substantial hepatic toxicity and intestinal irritation, highlighting the urgent need for optimized delivery strategies to maximize efficacy and minimize systemic toxicity.

### Structural characterization of SF-HA and IMSFs gels

**Figure 2:**
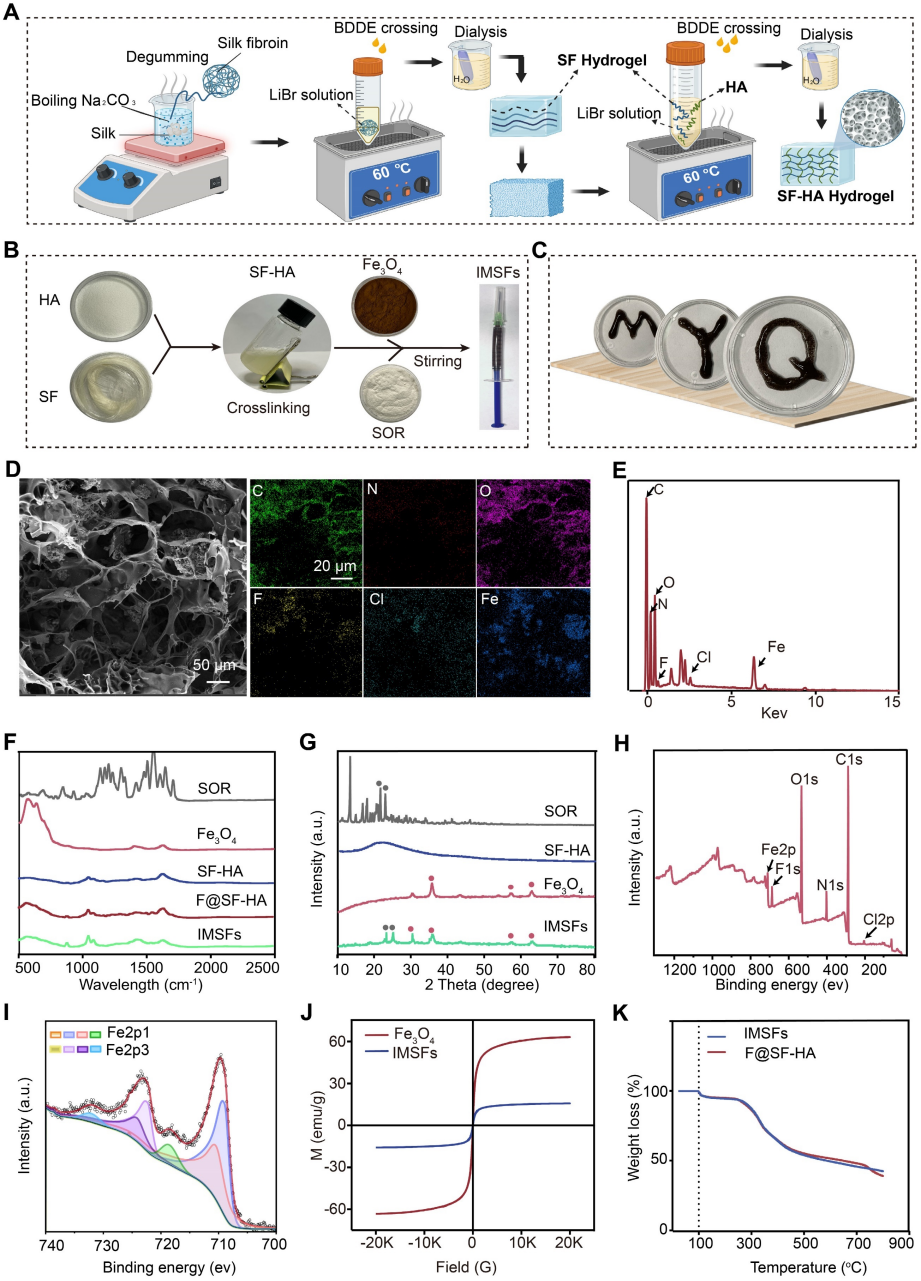
** SF-HA and IMSFs gel structure and characteristics.** (A) Schematic diagram of the preparation of SF-HA Gels and (B) IMSFs Gels. (C) Illustrations of the injectable IMSFs passing through a 1 mL standard syringe, along with images depicting various forms. (D) SEM photographs and their respective mapping images showing IMSFs with AMF exposure. (E) The energy spectrum of IMSFs. (F) FTIR spectra of SOR, Fe_3_O_4_, SF-HA, F@SF-HA, and IMSFs. (G) XRD spectra of SOR, Fe_3_O_4_, SF-HA, and IMSFs. (H) XPS spectra of IMSFs. (I) Fe2p XPS of IMSFs. (J) Magnetic hysteresis loop of IMSFs and Fe_3_O_4_ nanoparticles. (K) TGA curves of F@SF-HA and IMSFs.

To mitigate systemic toxicity and improve tumor-specific ferroptosis, we engineered injectable magnetic thermoresponsive silk fibroin-based hydrogels (IMSFs) for localized drug delivery. Silk fibroin-hyaluronic acid (SF-HA) hydrogels were synthesized using the FDA-approved crosslinker 1,4-butanediol diglycidyl ether (BDDE) (Figure 2A). This two-step fabrication method is straightforward, ensuring ease of operation and high crosslinking reliability suitable for biomedical applications. The resulting SF-HA scaffold exhibited minimal phase separation (Figure S2) and excellent structural stability over 20 d (Figure S3), surpassing traditional HA hydrogels that degrade rapidly. Live/dead assays further confirmed biocompatibility, essential for clinical applications (Figure S4). Subsequently, Fe₃O₄ nanoparticles and sorafenib powder were incorporated into the SF-HA scaffold, forming IMSFs without phase separation after 12 h of stabilization (Figure 2B), ensuring sufficient time for practical injection. The IMSFs gel displayed excellent injectability through a standard syringe (Figure S5) and readily conformed to irregular tumor shapes (Figure 2C), suggesting significant potential for minimally invasive TNBC treatment.

Scanning electron microscopy (SEM) confirmed the hydrogel's porous structure with uniformly dispersed Fe₃O₄ nanoparticles and sorafenib particles (Figure S6). Energy dispersive spectroscopy (EDS) mapping further validated their homogeneous distribution (Figure 2D and Figure S7). Post-AMF exposure, IMSFs retained structural integrity, with average mesopore diameter notably increasing (~2.787-fold increase), critical for magnetic-thermally responsive drug release, as confirmed by BET analysis. Quantitative EDS elemental analysis is presented in Figure 2E. Structural integrity of Fe₃O₄ and sorafenib during IMSFs synthesis was validated through XRD and FTIR analyses (Figure 2F-G), and XPS analysis confirmed no chemical interaction among components (Figure 2H). ICP-MS analysis demonstrated a high encapsulation efficiency (97.3%), confirming the hydrogel's excellent drug-loading capacity. Fe₃O₄ nanoparticles facilitated controlled Fe²⁺ and Fe³⁺ ion release, promoting intracellular iron overload and ROS generation, thereby enhancing ferroptosis (Figure 2I).

For effective magnetic hyperthermia therapy (MHT), adequate magnetic properties are crucial 39, 40. Magnetic hysteresis curves demonstrated that IMSFs exhibited soft magnetic behavior with low coercivity, suitable for robust magnetic hyperthermia therapy (MHT) upon AMF exposure (Figure 2J). Thermogravimetric analysis (TGA) confirmed IMSFs' thermal stability at temperatures relevant for MHT (< 50 °C) (Figure 2K).

In summary, the engineered IMSFs hydrogel exhibits robust structural integrity, injectability, and controlled drug release, underscoring its suitability as a minimally invasive therapeutic platform for TNBC.

### Magnetic hyperthermic properties of IMSFs

**Figure 3:**
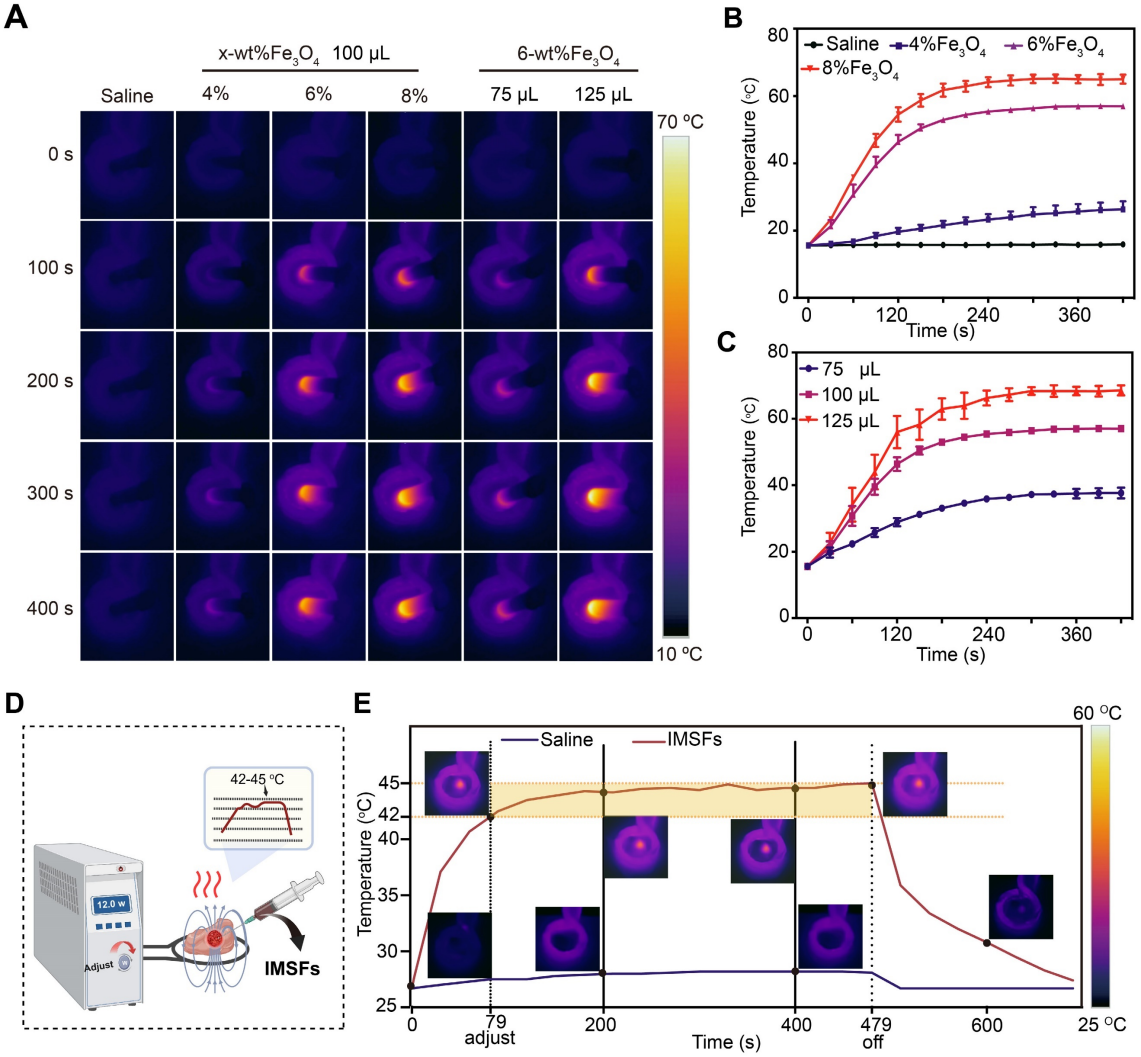
** Magnetic hyperthermia properties of IMSFs.** (A) *In vitro* infrared thermal images of IMSFs with different volumes and various mass fractions of Fe_3_O_4_ nanoparticles. (B) Corresponding quantitative temperature curves of saline and IMSFs (incorporated with varying fractions of mass of Fe_3_O_4_ nanoparticles) (n = 3). (C) Corresponding quantitative temperature curves of 6% Fe_3_O_4_- IMSFs with various volumes (n = 3). (D) Schematic diagram of an *in vitro* bovine liver therapy model for regulating power-stable magnetocaloric temperature. (E) 100 μL 6%Fe3O4-IMSFs was used to adjust the quantitative temperature curve of *in vitro* magnetothermal treatment of bovine liver with stable magnetocaloric power and the corresponding infrared thermal images.

To achieve controlled mild magnetic hyperthermia therapy (MTT) within the therapeutic temperature range of 42-45 °C, determining the optimal Fe₃O₄ loading ratio is crucial. Infrared thermal imaging (Figure 3A) and corresponding temperature curves confirmed that IMSFs rapidly and effectively elevated local temperatures under an AMF, while the physiological saline control exhibited no significant temperature increase. Comparative analysis of IMSFs with varying Fe₃O₄ loadings (4%, 6%, and 8%) revealed that 4% Fe₃O₄-IMSFs failed to reach the desired hyperthermic range, while both 6% and 8% Fe₃O₄-IMSFs exceeded 45 °C (Figure 3B). Notably, 8% Fe₃O₄-IMSFs caused excessive temperature to rise above 60 °C, which could lead to thermal damage to surrounding tissues. As the volume of 6% Fe₃O₄-IMSFs increased, the hyperthermic effect intensified proportionally (Figure 3C). However, when the injected volume reached 125 µL, the maximum temperature exceeded 60 °C again, making it unsuitable for mild hyperthermia applications. Based on these observations, 100 µL of 6% Fe₃O₄-IMSFs was identified as the optimal formulation for achieving controlled and effective MTT without excessive heating.

To simulate the therapeutic process of IMSFs in tumors, an ex-liver model was used. By adjusting the AMF power, the target temperature range of 42-45 °C was successfully maintained (Figure 3D), ensuring optimal therapeutic conditions while minimizing off-target thermal damage. As shown in Figure 3E, 100 µL of 6% Fe₃O₄-IMSFs exhibited excellent thermal stability, achieving a consistent and adjustable hyperthermic effect within tissues. This ability to finely control the temperature by adjusting AMF parameters highlights IMSFs' adaptability for precision-controlled tumor therapy.

After 400 s of magnetic hyperthermia, histological analysis revealed a well-defined necrotic zone, approximately 1 cm in diameter, characterized by pale liver tissue with varying degrees of coagulative necrosis (Figure S8). Notably, the sharp boundary between necrotic and healthy tissue suggests that hyperthermic damage was confined to the targeted area, with no significant thermal radiation effects on surrounding tissues. These findings validate IMSFs' ability to achieve tumor-specific thermal ablation while preserving adjacent healthy tissue, which is essential for clinical translation.

Based on these results, 100 µL of 6% Fe₃O₄-IMSFs was confirmed as the optimal formulation for MTT. Its controllable and localized heating capabilities make it an ideal candidate for combination therapies, where precise hyperthermia-induced ferroptosis can be synergistically integrated with drug release and immune activation strategies.

### Mild hyperthermia triggers Fe release and ROS accumulation

**Figure 4:**
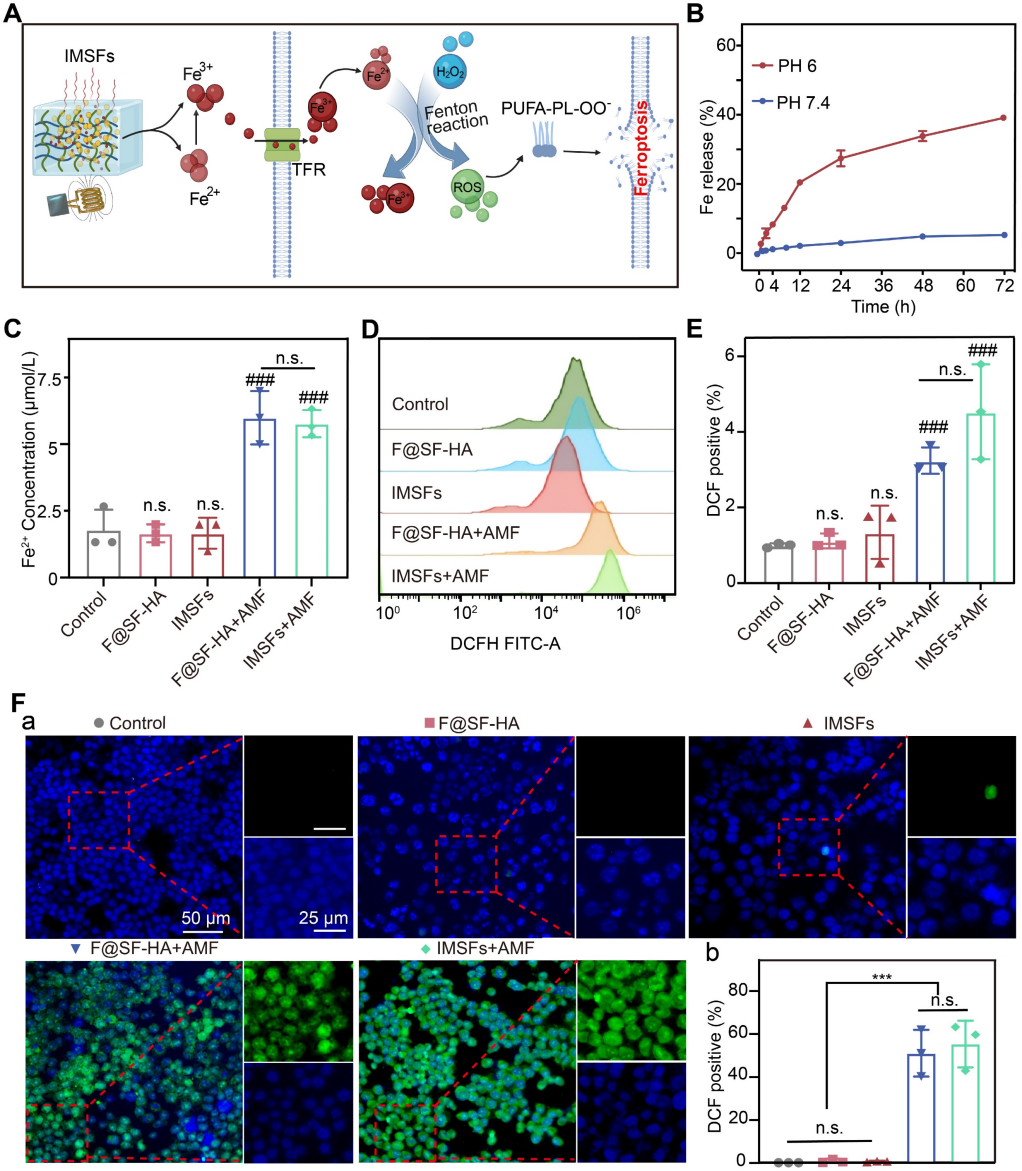
** Mild hyperthermia-triggered Fe release and ROS accumulation.** (A) Schematic diagram of the Fe Release and ROS Accumulation mechanism under the action of AMF. (B) Cumulative release of Fe from IMSFs at different pH levels. (C) Fe^2+^ concentration of 4T1 cells from various groups. (D)The FCM data illustrate the levels of intracellular ROS in 4T1 cells subjected to diverse treatments alongside (E) the associated quantitative assessments. (F) Representative DCF fluorescence imaging and the analysis of fluorescence intensity reflecting ROS levels in 4T1 cells following different treatment regimens. (The results are presented as mean values ± SDs, n = 3 per group. Statistical significance is indicated as follows: n.s. denotes non-significant differences, ###p < 0.001 when compared to the control groups, and ***p < 0.001.)

We investigated the mechanism by which mild hyperthermia facilitates Fe ion release and induces primary ferroptosis by analyzing the Fe-mediated reaction process and its synergistic effects. As anticipated, Fe₃O₄ nanoparticles within IMSFs released Fe²⁺/Fe³⁺ upon AMF exposure. The increase in extracellular Fe ion concentration promotes cellular Fe ion transport and uptake 41. Extracellular Fe²⁺ was oxidized to Fe³⁺ and internalized via transferrin-mediated endocytosis, followed by intracellular reduction to Fe²⁺, which catalyzed the Fenton reaction, generating ROS and initiating lipid peroxidation-driven ferroptosis (Figure 4A).

As shown in Figure S9, IMSFs in the HT+PH 6 group exhibited significantly increased Fe ion release compared to other conditions; minimal release occurred without heating, and heating alone resulted in less than 10% release. This demonstrates that IMSFs exhibit both pH- and heat-responsive release properties, with Fe ion release being more sensitive to acidic conditions, enhancing selective release in the acidic tumor microenvironment. Furthermore, under combined heat and acidic stimulation, Fe ion release was significantly enhanced, confirming that IMSFs possess controlled release capability under thermal stimuli. We simulated the tumor's local acidic environment and surrounding normal tissue, subjecting IMSFs to AMF exposure or no exposure to further validate their magnetic thermoresponsive release behavior. Results shown in Figure 4B and Figure S10 confirm that Fe dissolution in the acidic tumor microenvironment (pH 6) was significantly amplified, whereas minimal release occurred in the normal tissue environment (pH 7.4). AMF exposure further enhanced Fe ion release in acidic conditions, demonstrating the pH- and AMF-responsive Fe ion release from IMSFs. This dual-responsive behavior enables tumor-selective Fe ion accumulation and enhanced release under magnetic field control, mitigating toxicity risks to normal tissues.

Intracellular Fe²⁺ measurements revealed that AMF exposure significantly increased Fe²⁺ levels in both F@SF-HA and IMSFs groups, with no significant differences between the two (Figure 4C). This confirms a positive correlation between extracellular Fe ion concentration and intracellular Fe²⁺ levels. Magnetic hyperthermia is the primary driver of Fe ion release, with sorafenib exerting no interference with the Fe-mediated Fenton reaction. Additionally, SF-HA plays a crucial role in stabilizing nanoparticle retention within the hydrogel matrix. Since Fe overload promotes ROS generation through the Fenton reaction 42, 43, we measured intracellular ROS levels using DCFH. A significant increase in ROS was observed only in AMF-exposed cells, with the IMSFs-treated group showing a fourfold increase in ROS compared to controls (Figure 4E). Moreover, ROS accumulation correlated with Fe ion levels (Figure 4D-F), further supporting that Fe release from IMSFs effectively induces oxidative stress and ferroptosis. These results demonstrate that IMSFs, upon AMF stimulation, enable tumor-selective Fe ion release, promoting ROS-driven ferroptosis. The pH-sensitive, AMF-regulated Fe release mechanism ensures precise spatial and temporal control, laying the foundation for future integration with drug release and immune activation strategies.

### Efficacy of mutually synergistic therapy *in vitro*

**Figure 5:**
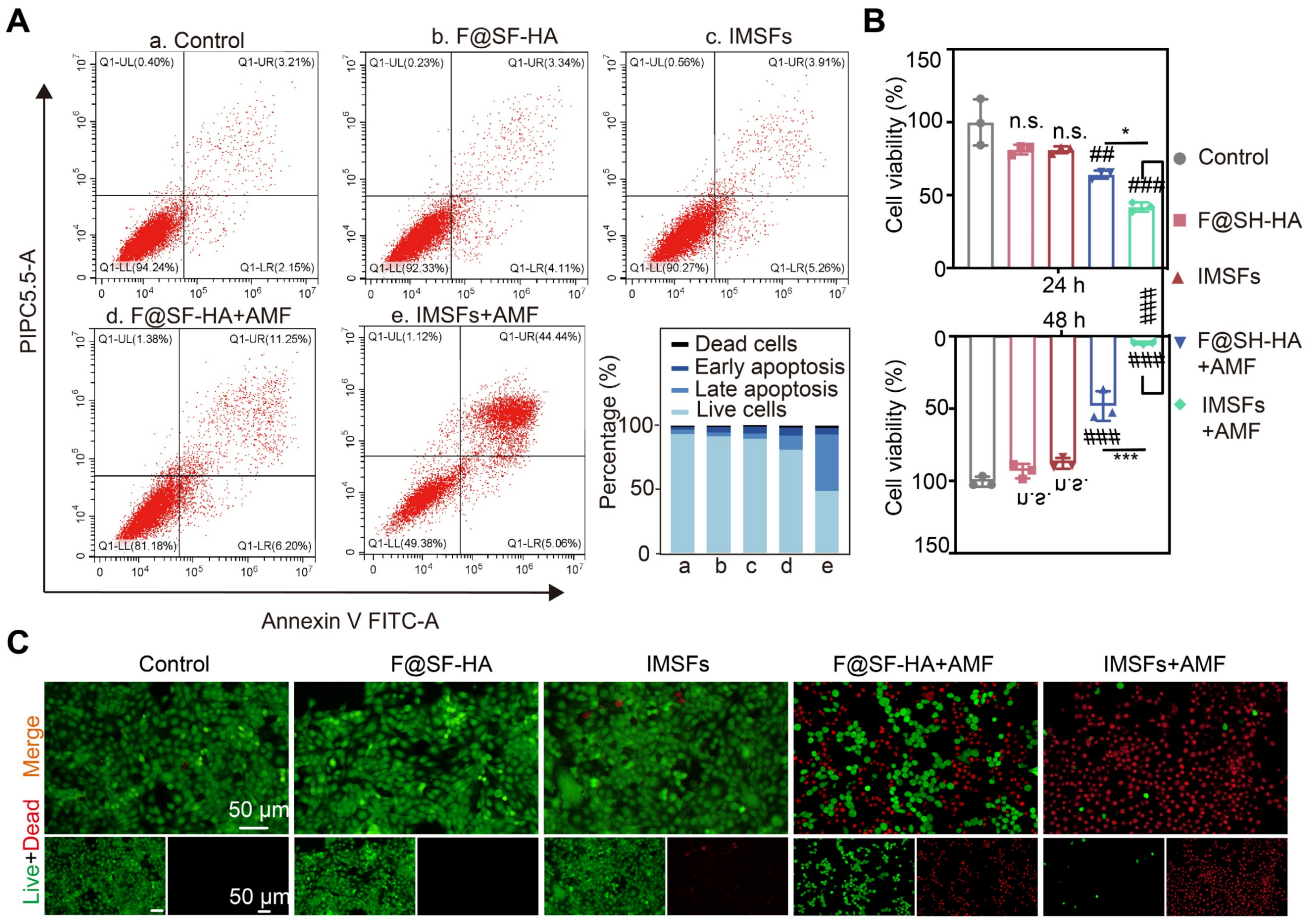
** Efficacy of mutually synergistic therapy *in vitro*.** (A) The apoptosis of 4T1 cells cocultured with PBS, F@SF-HA, IMSFs, F@SF-HA+AMF, and IMSFs+AMF for 24 h was measured and analyzed using flow cytometry. (B) The viability of 4T1 cells subjected to various treatments for 24 h and 48 h was evaluated using the CCK8 assay. (C) After 48 h of different treatments, fluorescence imaging captured live (green) and dead (red) 4T1 cells. (The results are presented as mean values ± SDs, n = 3 per group. Statistical significance is indicated as follows: n.s. denotes non-significant differences, ##p < 0.01, ###p < 0.001 when compared to the control groups, and *p < 0.05, ***p < 0.001 for the distinction between F@SF-HA +AMF and IMSFs+AMF groups.)

Effective anti-cancer activity and biocompatibility are fundamental prerequisites for the *in vivo* application of responsive, functionalized implants. The therapeutic window of IMSFs was defined by its on-demand activation profile. Without AMF exposure, IMSFs exhibited negligible cytotoxicity, with 4T1 cell viability remaining above 90% at both 24 and 48 h. However, upon AMF-triggered magnetic hyperthermia (400 s), a marked decline in cell viability was observed in the IMSFs group relative to the F@SF-HA group, with cell viability dropping to nearly zero at 48 h (Figure 5A-C). These results demonstrate that IMSFs are inherently biocompatible, as indicated by their minimal cytotoxicity in the absence of AMF exposure. Moreover, previous studies have shown that AMF exposure at this power level does not directly harm cells 44. The enhanced cytotoxicity observed in the AMF-exposed IMSFs group strongly suggests that the combination of magnetic hyperthermia and Fe-ion release leads to ROS accumulation. Additionally, the further reduction in viability may be attributed to sorafenib's synergistic effect within the IMSFs system. These findings highlight the excellent performance of IMSFs as an anti-cancer drug delivery system, underlining their tumor-selective lethality, which is a hallmark of precision ferroptosis therapy.

### Mechanisms of tumor suppression by IMSFs under AMF exposure

**Figure 6:**
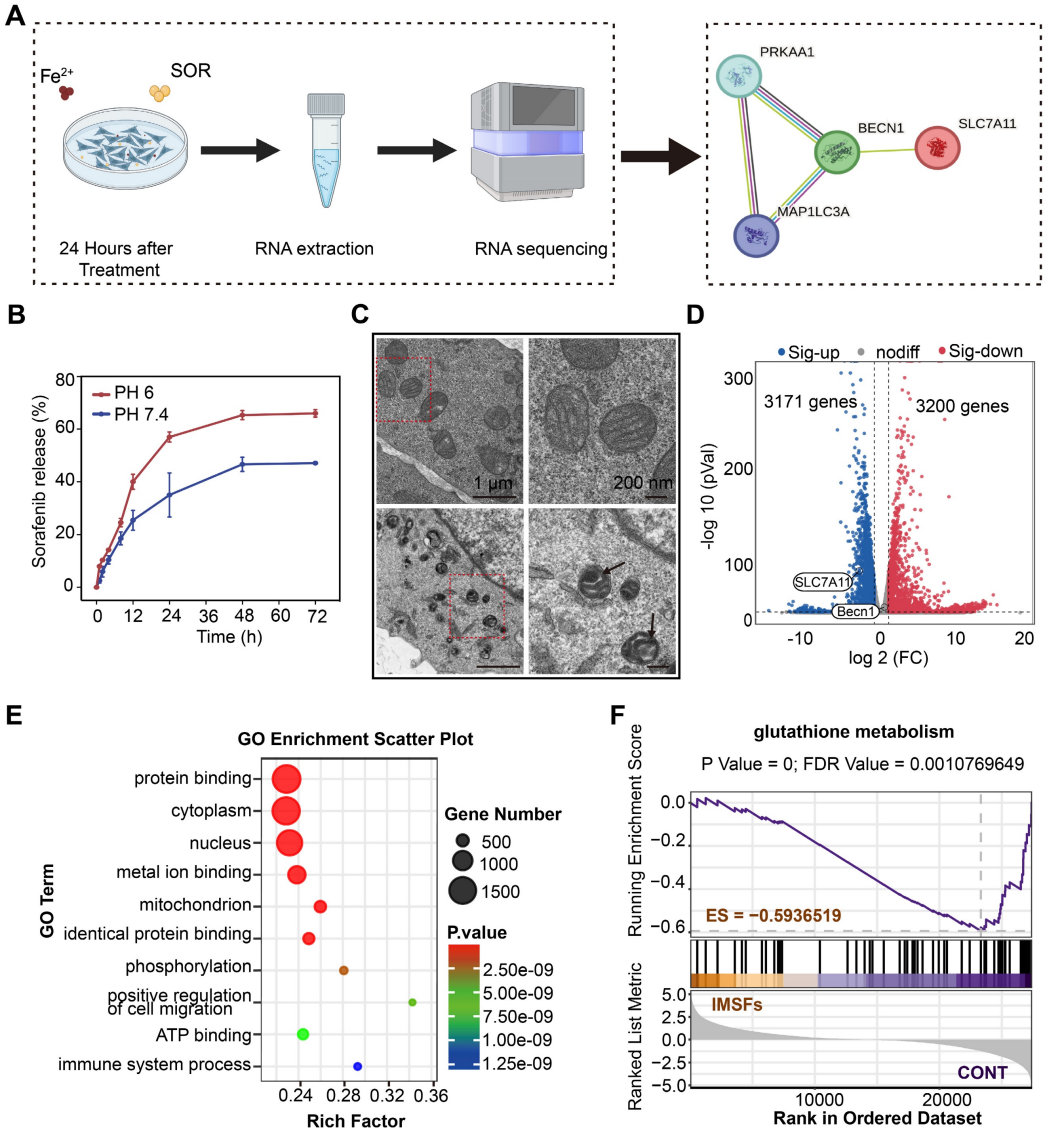
** Mechanisms of tumor suppression by IMSFs** under AMF Exposure**.** (A) Schematic diagram of the RNA sequence analysis of 4T1 cells from the Control and IMSFs+AMF groups. (B) Cumulative release of sorafenib from IMSFs at different pH levels (n = 3). (C) Comparative images of the transmission electron microscope of two groups of 4T1 cells after various treatments. (D) A volcano plot illustrates the differentially expressed genes between the Control and IMSFs+AMF groups. (E) Representative GO pathways associated with genes significantly differentially expressed between the Control and IMSFs+AMF groups. (F) The glutathione metabolism pathway is enriched in the IMSFs+AMF group.

To delineate the cascade antitumor mechanism of IMSFs and explore sorafenib's role in GPX4 suppression, we conducted transmission electron microscopy (TEM) imaging and transcriptomic sequencing on 4T1 cells treated with IMSFs under AMF exposure. As hypothesized, sorafenib release from IMSFs suppressed system Xc⁻ activity through the AMPK-Beclin1-SLC7A11 axis, amplifying ROS accumulation and exacerbating ferroptosis (Figure 6A). Controlled release assays of SOR (Figure 6B and Figure S11) showed that without AMF exposure, negligible release occurred, demonstrating the excellent magnetic thermoresponsive release ability of IMSFs, which ensures localized GPX4 suppression with enhanced tumor specificity and therapeutic safety. TEM imaging revealed classical ferroptotic morphology in IMSFs-treated cells, including mitochondrial shrinkage and membrane thickening (Figure 6C), directly linking iron-driven oxidative stress to the observed cell death phenotype 45. These morphological findings provided critical biological context for the subsequent transcriptomic data, reinforcing ferroptosis as the dominant cell death mechanism under IMSFs treatment.

Transcriptomic profiling identified 3,171 upregulated and 3,200 downregulated genes in IMSFs-treated cells compared to controls (Figure 6D). Notably, SLC7A11—a key regulator of GSH synthesis and sorafenib's GPX4 inhibitory target—was significantly downregulated. In contrast, Beclin1 expression remained unchanged, suggesting post-translational modulation via the Beclin1-SLC7A11 complex formation, rather than transcriptional suppression. While KEGG analysis did not directly enrich the ferroptosis pathway (Figure S12), Gene Set Enrichment Analysis (GSEA) confirmed significant suppression of glutathione metabolism (ES = -0.59365, p < 0.05; Figure 6F), aligning with system Xc⁻ dysfunction and GSH depletion. GO term enrichment further supported this mechanism, highlighting processes such as “protein binding” (complex formation), “metal ion binding” (Fenton reaction-mediated oxidation), “phosphorylation” (kinase signaling), and “organelle organization” (mitochondrial damage) (Figure 6E).

The lack of KEGG ferroptosis pathway enrichment likely reflects the multifactorial regulation of ferroptosis, involving lipid peroxidation, iron dysregulation, and antioxidant system failure. Integrating these findings, we propose that IMSFs induce ferroptosis via the AMPK-Beclin1-SLC7A11 axis. Sorafenib, a known AMPK activator, disrupts mitochondrial complex I, elevating AMP/ATP ratios and triggering AMPK phosphorylation 46, 47. Activated AMPK phosphorylates Beclin1, promoting its binding to SLC7A11, functionally inactivating system Xc⁻—independent of transcriptional regulation—to block cystine uptake and deplete GSH 33. Concurrently, Fe₃O₄-derived Fe²⁺ amplifies lipid peroxidation via Fenton reactions, synergizing with GPX4 inactivation (due to GSH loss) to potentiate membrane damage. SLC7A11 downregulation may arise from post-translational destabilization or altered subcellular localization following Beclin1-SLC7A11 complex formation. Additionally, GO term enrichment in phosphorylation and protein binding further supports AMPK activation and complex assembly.

Collectively, these data establish that sorafenib exerts localized GPX4 suppression through the AMPK-Beclin1-SLC7A11 axis, which serves as the central mechanism driving IMSFs-induced ferroptosis.

### Sorafenib's GPX4-inhibitory effect and mechanistic validation

**Figure 7:**
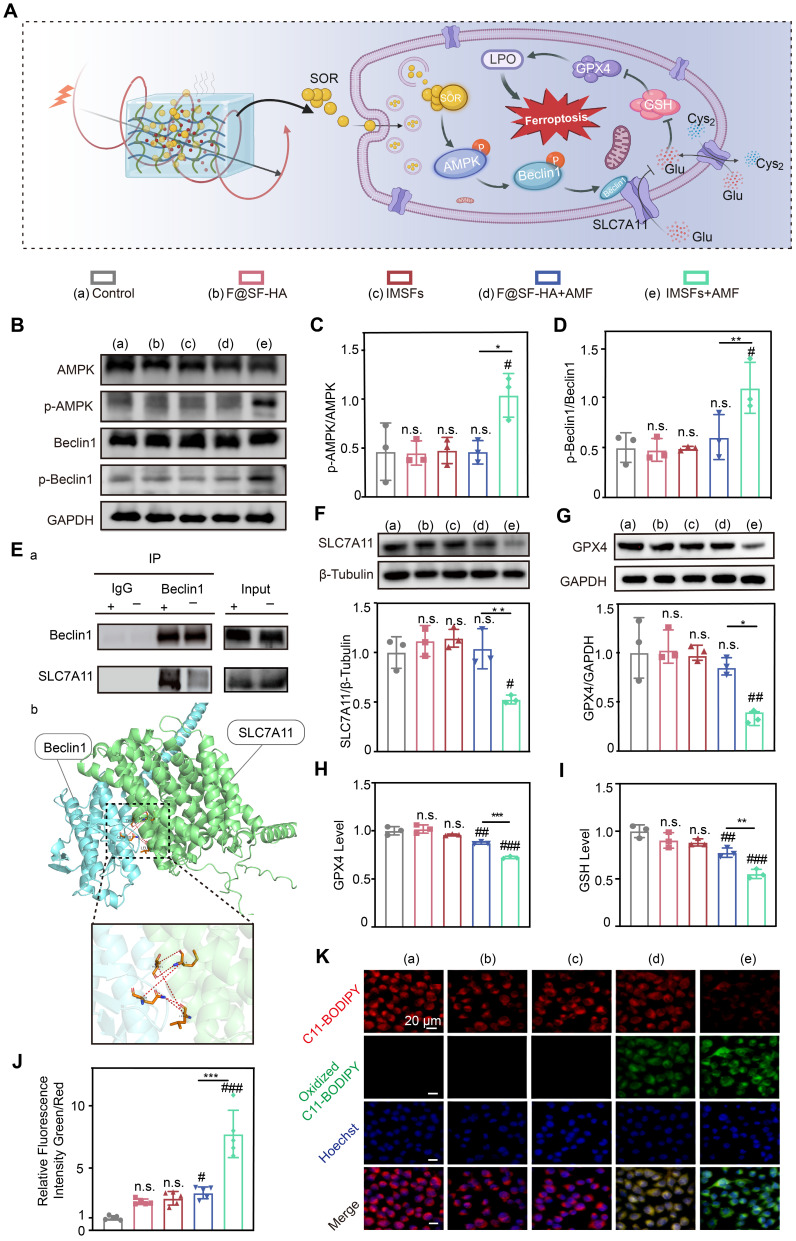
** Sorafenib's GPX4-inhibitory effect and mechanistic validation.** (A) Schematic diagram of the mechanism of iron death induced by sorafenib release. (B) Representative western blot images of p-AMPK, AMPK, p-Beclin1, Beclin1, and GAPDH in the indicated groups. Quantitative analyses of the (C) p-AMPK/AMPK and (D) p-Beclin1/Beclin1 ratios. (E) Co-immunoprecipitation (Co-IP) demonstrates the Beclin1-SLC7A11 complex and molecular docking analysis between BECN1 and SLC7A11. (F) Expression of SLC7A11 protein in different groups. (G) Expression of GPX4 protein in different groups. (H) The Glutathione peroxidase assay kit assessed the GPX4 concentration of 4T1 cells from different groups. (I) Intracellular GSH and (J) LOP contents of 4T1 cells in different treatment groups. (K) LOP fluorescence images of 4T1 cells after different treatments. (The results are presented as mean values ± SDs, n = 3 per group. Statistical significance is indicated as follows: n.s. denotes non-significant differences, #p < 0.05, ##p < 0.01, ###p < 0.001when compared to the Control groups, and *p < 0.05, **p < 0.01, ***p < 0.001 for the distinction between F@SF-HA+AMF and IMSFs+AMF groups.)

Transcriptomic sequencing revealed that IMSFs locally suppress GPX4 primarily through magnetothermal-responsive sorafenib release, which activates AMPK and induces Beclin1 phosphorylation, promoting Beclin1-SLC7A11 complex formation, leading to System Xc⁻ inactivation. This cascade impairs cystine-glutamate exchange, depletes GSH, reduces GPX4 levels, and disrupts oxidative defense, ultimately accelerating ferroptosis in 4T1 cells (Figure 7A). To validate these findings, we compared five experimental groups: untreated control, Fe@SH-HA, IMSFs, Fe@SH-HA+AMF, and IMSFs+AMF. Western blot analysis confirmed that AMF-exposed IMSFs induced significant AMPK and Beclin1 phosphorylation compared to other groups (Figure 7B-D), aligning with transcriptomic predictions. To further examine Beclin1's role in SLC7A11 regulation, molecular docking simulations identified potential interaction sites between Beclin1 and SLC7A11, which were subsequently validated through co-immunoprecipitation (Co-IP) assays, confirming direct Beclin1-SLC7A11 complex formation (Figure 7E). These results demonstrate that AMF-triggered sorafenib release activates AMPK phosphorylation, drives Beclin1 phosphorylation, and facilitates Beclin1-SLC7A11 complex assembly, solidifying the AMPK-Beclin1-SLC7A11 axis as the core regulatory pathway.

Notably, AMF-exposed IMSFs markedly downregulated GPX4 and SLC7A11 protein levels (Figure 7F-G), a trend corroborated by GPX4 activity assays and GSH quantification (Figure 7H-I), indicating severely compromised oxidative defense in the IMSFs+AMF group. This underscores sorafenib's critical role in inhibiting System Xc⁻, as SLC7A11 dysfunction directly impairs GSH synthesis and GPX4 activity, pivotal steps in ferroptosis induction. The controlled release of sorafenib from IMSFs under AMF ensures localized GPX4 suppression, minimizing off-target effects while enhancing therapeutic precision. Using the lipid peroxidation (LPO) sensor C11-BODIPY, we observed an 8-fold increase in lipid ROS levels in IMSFs+AMF-treated cells versus controls (Figure 7J), with fluorescence microscopy confirming intense green signal accumulation (Figure 7K). In contrast, F@SH-HA + AMF induced only a 3-fold ROS increase, demonstrating sorafenib's indispensable role in amplifying ferroptosis beyond mere iron overload. These data indicate that sorafenib-mediated GPX4 suppression synergizes with ROS accumulation to amplify lipid peroxidation, creating a cascade effect that escalates ferroptotic damage beyond additive effects.

### Antitumor efficacy of SF-platform: IMSFs-mediated MDT combined with TAT-Beclin1 *in vivo*

**Figure 8:**
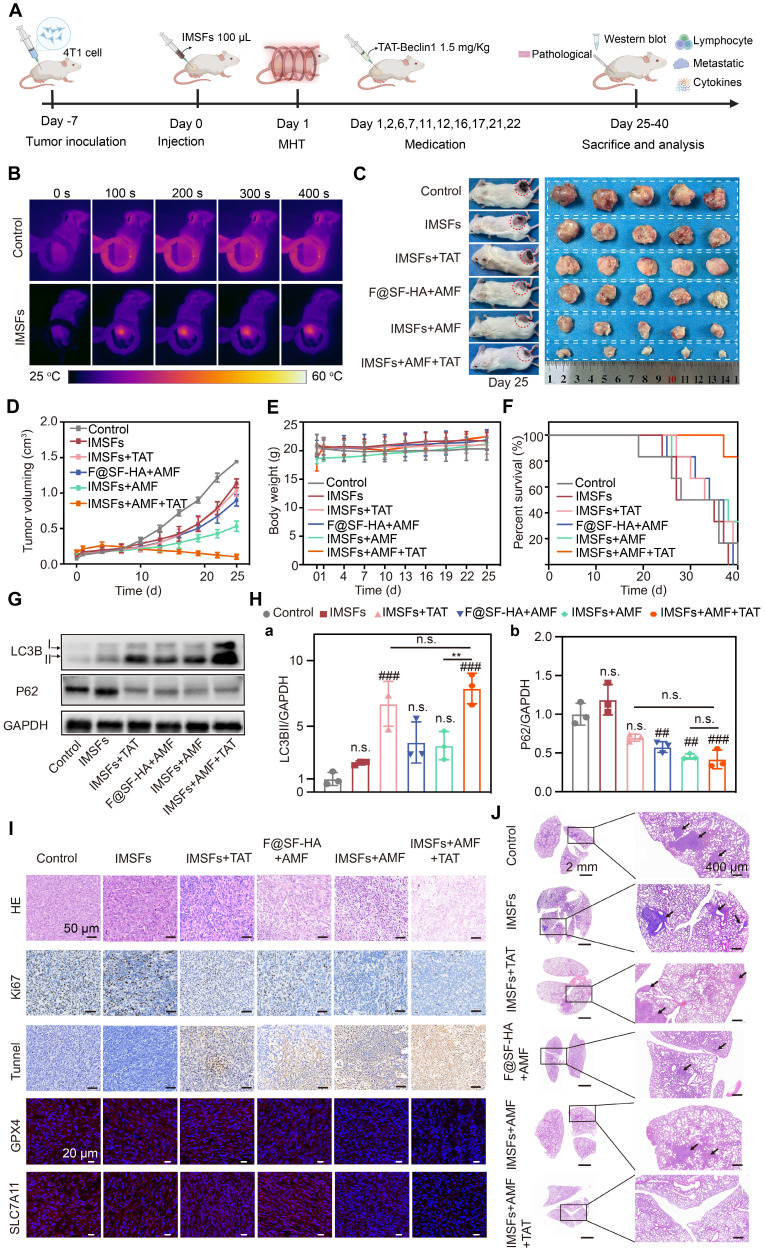
** Antitumor efficacy of SF-platform: IMSFs-mediated MDT combined with TAT-Beclin1 *in vivo*.** (A) Schematic timeline of the *in vivo* study. (B) Infrared thermal images depicting 4T1 tumor-bearing mice following intratumoral administration of saline or IMSFs under AMF exposure. (C) Representative images of excised 4T1 tumors and corresponding tumor-bearing mice after 25 d of treatment (n = 5). (D)Tumor volume progression in response to different treatment regimens (n = 5). (E) Body weight variations of 4T1 tumor-bearing mice across different treatment groups (n = 5). (F) Survival rates of tumor-bearing mice subjected to different therapeutic interventions (n = 5). (G) Expression of LC3II and P62 demonstrated by WB (n = 3). (H) Quantitative analyses of the LC3II and p62 levels (n = 3). (I) H&E staining, IF staining of Ki67 and TUNEL, and IHC staining of GPX4 and SLC7A11. (J) H&E staining of lung sections. (The results are presented as mean values ± SDs, n = 5/n = 3 per group. Statistical significance is indicated as follows: n.s. denotes non-significant differences, ##p < 0.01, ###p < 0.001when compared to the Control groups, and **p < 0.01 for the distinction between IMSFs+AMF and IMSFs+AMF+TAT groups.)

After validating the *in vitro* antitumor efficacy of IMSFs, we established orthotopic 4T1 mammary tumor models in BALB/c mice to assess their therapeutic potential *in vivo*, particularly when combined with TAT-Beclin1 (Figure 8A). Mice were randomized into five groups: Control, IMSFs alone, IMSFs+TAT, F@SF-HA+AMF, IMSFs+AMF, and IMSFs+AMF+TAT. Upon reaching tumor volumes of 100-120 mm³, intratumoral injections of F@SF-HA or IMSFs were administered, followed by AMF exposure. Infrared thermography confirmed localized tumor heating to 44 °C (maintained at 42-45 °C for 6 min) (Figure 8B and Figure S13), ensuring precise magnetothermal-triggered sorafenib release while minimizing heat radiation-induced damage to surrounding tissues. As shown in Figure 8C-D and Figure S14-15, the IMSFs-only treatment group exhibited minimal tumor growth inhibition, and the IMSFs+TAT group showed similar effects. In contrast, the F@SF-HA+AMF group resulted in less than 50% inhibition of tumor growth. However, the IMSFs+AMF+TAT group achieved the most potent suppression, with near-stagnant tumor progression and occasional volume reduction. These results suggest that autophagy alone has limited tumor suppression effects, while standalone MHT exerts limited antitumor effects, highlighting the necessity for combinatorial strategies. The suboptimal performance of IMSFs+AMF *in vivo* may stem from the tumor microenvironment's complexity and heterogeneity, which hampers full activation of the AMPK-Beclin1-SLC7A11 axis, coupled with gradual drug depletion, limiting sustained efficacy. The additional injection of TAT-Beclin1 synergistically enhanced the ferroptosis effect of IMSFs. As a membrane-permeable peptide, TAT-Beclin1's additional injection can avoid the impact of magnetic hyperthermia on its activity, showing excellent tumor penetration, facilitating Beclin1 activation, and compensating for insufficient endogenous Beclin1 activity in heterogeneous tumor regions. Furthermore, as an autophagy activator, TAT-Beclin1 likely amplifies ferroptosis by enhancing autophagic flux, thereby synergistically contributing to antitumor effects when combined with IMSFs.

Notably, no significant changes in body weight were observed across groups (Figure 8E and Figure S16), indicating high biosafety and biocompatibility of IMSFs and TAT-Beclin1 *in vivo*. Survival analysis further confirmed the therapeutic advantage of the combined treatment, with the IMSFs+AMF+TAT group exhibiting the highest 40-d survival rate (Figure 8F).

To validate TAT-Beclin1's effect on tumor autophagy promotion, we analyzed autophagic markers (LC3BII and p62) in tumor lysates. Results showed significantly elevated LC3BII expression and reduced p62 levels in both IMSFs+TAT and IMSFs+AMF+TAT groups (Figure 8G-H). An increase in LC3BII typically indicates autophagosome formation, while a decrease in the autophagic substrate p62 suggests enhanced autophagic flux 48. Thus, these results indicate that TAT-Beclin1 injection primarily enhances autophagic flux.

Histopathological assessment (H&E, Ki67, TUNEL) and oxidative defense markers (GPX4, SLC7A11) were analyzed (Figure 8I and Figure S17). The IMSFs+AMF+TAT group induced severe necrosis (H&E), maximal apoptosis (TUNEL), and significant proliferation inhibition (Ki67), surpassing other groups. Immunofluorescence confirmed synergistic GPX4 and SLC7A11 downregulation, suggesting that TAT-Beclin1 enhances autophagic flux while suppressing the Xc⁻ system, thus amplifying oxidative defense disruption and ferroptosis effects. This functionality is mediated by the further activation of Beclin1 in the IMSFs pathway, promoting the formation of the Beclin1-SLC7A11 complex 33. Additionally, the temporal control of TAT-Beclin1 injection after IMSFs treatment prevents premature ROS depletion by autophagy, ensuring the synergistic effect of autophagy and ferroptosis in the antitumor response.

Upon completion of the treatment regimen, pulmonary tumor nodules were assessed to evaluate the effect of IMSFs on tumor metastasis. At the end of treatment, digital imaging of Bouin's solution-stained lungs revealed minimal metastatic nodules in the IMSFs+AMF+TAT group, whereas the Control and IMSFs-alone groups exhibited extensive lung metastases (Figure S18). H&E staining confirmed these findings (Figure 8J). This result underscores the high metastatic tendency of TNBC, attributed to its immunosuppressive microenvironment. While ferroptosis is known to be immunogenic, our data suggest that IMSFs alone were insufficient to significantly remodel the immunosuppressive tumor microenvironment of this “cold tumor.” However, the SF-platform significantly suppressed tumor metastasis, suggesting that TAT-Beclin1 may help reverse the tumor's immunosuppressive microenvironment, thereby providing an additional advantage in inhibiting metastasis.

### Injectable SF-platform therapy induces ICD activation

Ferroptosis elicits antitumor immunity by releasing damage-associated molecular patterns (DAMPs), including high-mobility group box 1 (HMGB1) and calreticulin (CRT). CRT exposure on dying cells serves as an “eat-me” signal that promotes phagocytosis by antigen-presenting cells (APCs), while HMGB1 engages Toll-like receptor 4 (TLR4) on dendritic cells (DCs) to stimulate proinflammatory cytokine production and tumor antigen cross-presentation 49-51. These events drive DC maturation, subsequent naïve T cell priming, and cytotoxic T lymphocyte (CTL) differentiation, forming a cascade that activates adaptive immunity and amplifies immunogenic cell death (ICD).

**Figure 9:**
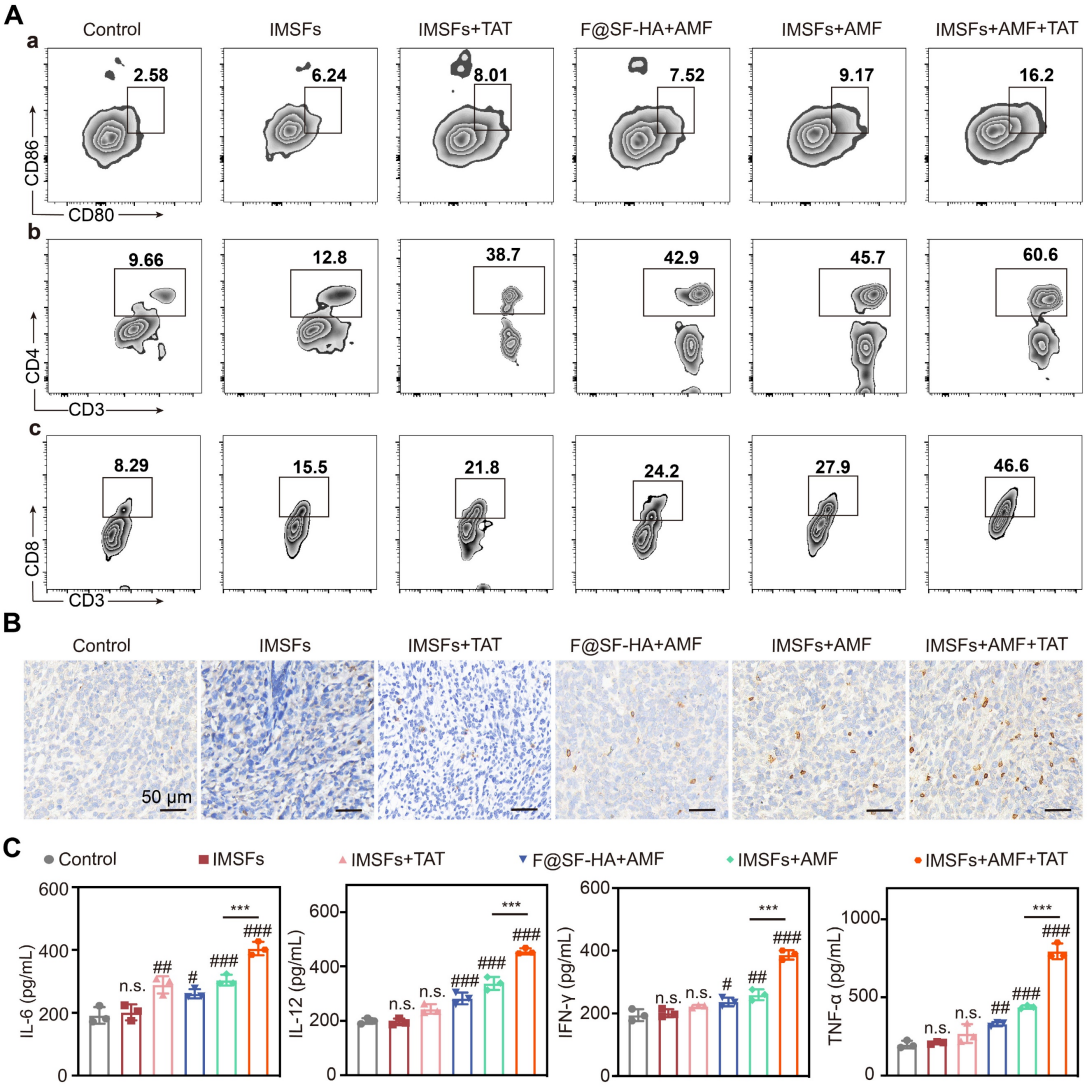
**Injectable SF-platform therapy induces ICD activation.** (A) FCM diagrams and quantification analysis of DC maturation and CTLs in the draining LNs adjacent to the primary tumors. (B) IF staining of CD8^+^. (C) The levels of TNF-α, IFN-γ, IL-6, and IL-12 in the serum of mice were measured by ELISA. (The results are presented as mean values ± SDs, n = 3 per group. Statistical significance is indicated as follows: n.s. denotes non-significant differences, #p < 0.05, ##p < 0.01 and ###p < 0.001 when compared to the Control groups, and ***p < 0.001 for the distinction between IMSFs+AMF and IMSFs+AMF+TAT groups.)

As ICD activation plays a pivotal role in suppressing metastasis and recurrence, we investigated its *in vivo* induction by analyzing immune cells from tumors, draining lymph nodes (LNs), and serum cytokines across treatment groups. Flow cytometry revealed that IMSFs+AMF+TAT induced maximal DC maturation. In contrast, IMSFs+AMF exhibited moderate effects, and other groups showed negligible results. Similarly, this combinatorial regimen triggered robust CD4⁺ and CD8⁺ T cell infiltration and differentiation, outperforming all monotherapies (Figure 9A and Figure S19). These results demonstrate that IMSFs alone fail to activate antitumor immunity, while AMF-mediated hyperthermia and TAT-Beclin1-mediated autophagy provide the necessary immunostimulation by enhancing T-cell activation. The limited DC maturation in IMSFs+AMF-treated tumors highlight the need for TAT-Beclin1 to overcome the immunosuppressive tumor microenvironment, likely compensating for Beclin1 activation in hypoxic regions.

ELISA quantification confirmed that IMSFs+AMF+TAT elicited the highest serum levels of TNF-α, IFN-γ, IL-6, and IL-12 (Figure 9C)—key cytokines driving antitumor immunity. This SF-platform synergizes with hyperthermia, ferroptosis, and autophagy to enhance ICD, thereby reversing the immunosuppressive microenvironment through tripartite activation of immune cell maturation, differentiation, and secretory functions. Notably, IFN-γ secreted by CD8⁺ T cells can suppress System Xc⁻ activity by inhibiting the expression of SLC7A11, exacerbating iron-dependent lipid peroxidation in tumor cells 52—a mechanism corroborated by our findings of elevated IFN-γ levels and CD8⁺ T cell infiltration in the combinatorial group (Figure 9B-C). In synergy with autophagy, this immune-mediated enhancement of ferroptosis amplifies the immune activation effect, forming a cascade of “ferroptosis-autophagy-immunity-ferroptosis.” This self-reinforcing feedback loop continuously targets and suppresses the Xc⁻ system, ultimately inducing a complete collapse of oxidative defenses in TNBC cells, ensuring sustained antitumor effects.

Importantly, this therapeutic synergy was achieved without systemic toxicity, as evidenced by normal serum biochemistry (Figure S20) and minimal histopathological changes in major organs (Figure S21). The localized action of IMSFs prevented off-target effects typically associated with oral sorafenib. These results highlight the SF platform's exceptional therapeutic efficacy and superior biosafety, with significant potential for clinical translation in TNBC management.

## Conclusion

In this study, we developed an injectable SF-platform (IMSFs-mediated MDT combined with TAT-Beclin1), a novel therapeutic strategy designed to induce a temporally controlled cascade of ferroptosis, autophagy, and immune activation for tumor therapy. This platform enables precise mild hyperthermia and localized drug release through AMF regulation, where the localized GPX4 suppression effect (via sorafenib release) synergizes with ROS accumulation (via Fe ion release) to initiate ferroptosis. Subsequently, TAT-Beclin1 injection activates Beclin1, enhancing autophagy and amplifying ferroptosis while simultaneously triggering immunogenic cell death (ICD). This results in a ferroptosis-autophagy-immune cascade, leading to the collapse of oxidative defense and driving sustained antitumor effects.

This integrated approach represents a novel paradigm for cancer therapy, offering targeted, precise, and time-controlled treatment with strong potential for clinical translation, particularly for refractory tumors. By harnessing iron-mediated oxidative stress, autophagy activation, and immune activation, this strategy provides a powerful tool for overcoming tumor resistance and metastasis.

## Materials and Methods

### Materials

Silk was supplied by Xiehe Silk Co., Ltd. (China). 1,4-Butanediol diglycidyl ether (BDDE) and hyaluronic acid (HA) were purchased from Macklin (Shanghai, China). Lithium bromide (LiBr) was obtained from Aladdin. (Shanghai, China). Fe_3_O_4_ nanoparticles (Fe_3_O_4_) were supplied by Aike Reagent (China). Sorafenib (SOR) was provided by Selleckchem (USA). TAT-Beclinl was obtained from Sigma-Aldrich (USA). In this study, all chemicals are analytical grade and stored under the manufacturer's guidelines.

### Establishment of a breast tumor model using 4T1 cells in BALB/c mouse

4T1 breast tumor cells were purchased from Sunncell (Wuhan, China) and cultured in a complete RPMI-1640 medium (Procell, China) at 37 °C with 5% carbon dioxide (CO_2_).

The *in vivo* experiments were approved by the Animal Experimentation Ethics Committee of The First Affiliated Hospital of Chongqing Medical University (CQMU). Female BALB/c mice (4-6 weeks old, 14-16 g) were purchased from CQMU and housed at the Animal Experiment Center. The mice were acclimatized for a week under standard laboratory conditions. On day -7, 4T1 breast tumor cells (1 × 10^6^ cells) were subcutaneously implanted in the backside region of the mouse to build xenograft tumor models. Once the tumors reached a volume of 100 mm³, these animals were assigned to subsequent experimental procedures.

### Oral efficacy and toxicity study of sorafenib

For the early-stage sorafenib study, on the initial day, the mice were allocated randomly into 2 separate Groups (n = 3): Control Group (CON): Received an equal volume of the vehicle (diluted 1:1: 8 DMSO/Cremophor EL/PBS) with sterile water. Sorafenib Group (SOR): Gavage administration of sorafenib at 30 mg/kg per day 53.

Throughout the treatment regimen, tumor volumes and body weights of the mice were recorded every 3 d. The following formula was used to calculate tumor volume. (volume = length × width^2^/2)

ALL mice were sacrificed 14 d post-treatment, blood and organ samples were harvested for toxicity evaluation.

### Preparation of silk hydrogels

Silk hydrogels were prepared following a previously described procedure 54. Briefly, silk was boiled for 30 min in an aqueous Na_2_CO_3_ solution (0.5%, w/v). The resulting product was thoroughly rinsed with deionized (DI) water and air-dried overnight in a fume hood to obtain degummed silk fibers.

Degummed silk fibers were thoroughly dissolved in a 9.3 M lithium bromide (LiBr) solution at 60 °C for 2 h. The silk solution was mixed with BDDE and incubated at 60 °C for 3 h. The obtained hydrogels were purified by DI water for 3 d, changing the water 15 times.

### Preparation of SF-HA hydrogels

The prepared silk hydrogels were lyophilized. Lyophilized silk hydrogels (1 g) and HA (1 g) were thoroughly dissolved in 9.3 M LiBr solution at 60 °C for 3 h. The dissolved solution was supplemented with BDDE and incubated at 60 °C for 3 h. The crosslinked hydrogels were washed with DI water for 3 d, with the water being changed 15 times.

### SF-HA hydrogels cellular compatibility assays and stability evaluation

50 μL of hydrogel was placed in 24-well plates, and 4T1 cells (5 × 10^3^) were seeded on the hydrogels for 48 h to evaluate the cellular compatibility of the hydrogels. The Control group, which consisted of a culture system without hydrogels, was included for comparison. Then, Calcein-AM and PI (15:5 μL) were added to stain live cells (green) and dead cells (red) and observed using a fluorescence microscope (CKX53).

The stability of the SF-HA hydrogels was assessed by placing them in glass vials at room temperature for one week to check for phase separation. The hydrogels were then lyophilized and weighed, after which they were immersed in pH 7.4 PBS to monitor the potential degradation of the lyophilized blocks. On day 20, the hydrogels were removed, lyophilized again, and weighed to evaluate mass changes.

### Synthesis and characterization of the F@SF-HA and IMSFs

As shown in Table 1, Fe₃O₄ and sorafenib (SOR) were incorporated into the SF-HA hydrogel at varying mass ratios to fabricate IMSFs hydrogels, while F@SF-HA hydrogels did not contain sorafenib. Using liquid nitrogen and fracturing under brittle conditions, we obtained cross-sectional samples of these hydrogels. The morphologies of the particles and hydrogels were examined via scanning electron microscopy (SEM) using a TESCAN MIRA LMS system, and BET was analyzed using the ASAP 2460 Version 3.01 system, both with and without alternating magnetic field (AMF) exposure.

Elemental distribution was assessed by energy-dispersive spectroscopy (EDS) under the same SEM parameters. Fourier-transform infrared (FTIR) spectroscopy (Nicolet iS20) was used to record the infrared spectra of sorafenib, Fe₃O₄, SF-HA, F@SF-HA, and IMSFs hydrogels. Structural analysis was performed using X-ray diffraction (XRD) (Akishima, Japan), and X-ray photoelectron spectroscopy (XPS) measurements were conducted using a Thermo Scientific K-Alpha instrument. Encapsulation efficiency was determined using inductively coupled plasma mass spectrometry (ICP-MS, Agilent 5800 OES). Magnetic hysteresis loop measurements were carried out using a Lakeshore 7404 instrument. Thermal stability was analyzed via thermogravimetric analysis (TGA) using a Mettler TGA/DSC3^+^ instrument. AMF exposure was induced using an AMF generator (SPG400K2-10) with the following parameters: coil length: 1 cm, coil turns: 2, coil diameter: 3 cm, frequency: 626 kHz, output current: 28.6 A, field strength: 5.72 kA/m. All power settings were maintained within biologically safe limits to induce the magnetothermal effect.

### *In vitro* magnetothermal efficiency of various forms of IMSFs

Fe₃O₄ nanoparticles were incorporated at varying mass fractions (4%, 6%, and 8%), and multiple volumes of IMSFs (75 μL, 100 μL, and 125 μL) were injected into isolated bovine liver tissue and phosphate-buffered saline (PBS) to assess the magnetothermal efficiency of IMSFs exposed to the same AMF power. Temperature changes were monitored using a far-infrared thermometer (FOTRIC225), and thermal imaging data were analyzed using AnalyzIR 7.1 software. Each experimental condition was performed in triplicate to ensure the reproducibility of results.

To highlight stable magnetothermal heating in the range of 42-45 °C, the optimal IMSF composition and volume were selected. In subsequent experiments, the temperature was monitored with the far-infrared thermometer, and the magnetic field strength was adjusted via the AMF generator to maintain a steady treatment temperature within the 42-45 °C range.

### Investigation of Fe ion release sensitivity

200 μL of IMSFs gel was transferred into dialysis tubing and placed in a 50 mL centrifuge tube containing 20 mL of physiological saline solution. The release system was treated according to the following groups: Control: pH 7.4, 25 °C; HT group: pH 7.4, 45 °C; pH 6 group: pH 6, 25 °C; HT+pH 6 group: pH 6, 45 °C. The dissolution system was mechanically shaken in an incubator at 120 rpm for 48 h. At predetermined intervals, release medium samples were collected for analysis, and fresh medium was added to replace the samples. The concentration of released Fe ions in the supernatant was analyzed using ICP-MS (Agilent 720ES).

### Evaluation of mild hyperthermia-triggered sorafenib and Fe²⁺/Fe³⁺ release

Prepared 100 μL IMSFs gel was carefully transferred into a 15 mL centrifuge tube, with 10 mL of PBS solution adjusted to either pH 7.4 or pH 6 and containing 1% (w/v) Tween 80 and lysozyme as the release medium. The system was exposed to AMF for 400 s. Following AMF exposure, the dissolution system was mechanically shaken in an incubator at 37 °C and 120 rpm for 72 h. At predetermined intervals, release medium samples were collected for analysis, and fresh medium was added to replace the samples. The concentration of released sorafenib in the supernatant was analyzed using a UV-vis spectrophotometer.

Additionally, total iron ion concentrations were determined after reducing Fe³⁺ to Fe²⁺ with sodium sulfite. 200 μL IMSFs gel was transferred into dialysis tubing and placed in a 50 mL centrifuge tube containing 20 mL of physiological saline solution adjusted to either pH 7.4 or pH 6. The release conditions and treatments were the same as described above. The iron ion concentrations were measured using ICP-MS (Agilent 720ES).

### Measurement of cellular iron levels

Intracellular ferrous ion concentrations were evaluated using a ferrous ion detection kit across several groups: Control, F@SF-HA, IMSFs, F@SF-HA+AMF, and IMSFs+AMF. F@SF-HA or IMSFs hydrogels, as indicated in the experimental groups, were co-cultured with 4T1 cells. In the AMF groups, the culture temperature was maintained within the 42-45 °C range for 400 s. After treatment, all experiments were conducted at 37 °C.

### Cellular ROS assessment

Cells of the 4T1 line were placed into 12-well plates and allowed to culture for 24 h. Following the administration of different treatments, the cells were rinsed using PBS. Subsequently, they were subjected to a 30 min incubation with DCFH-DA (Beyotime, China) followed by a 5 min staining with Hoechst33342 (Beyotime). The intracellular ROS levels were evaluated next, utilizing a fluorescence microscope and flow cytometry (FCM) analysis.

### *In vitro* therapeutic efficacy of IMSF

4T1 cells were seeded into 96-well plates at a density of 1 × 10^4^ cells per well and treated with various treatments for 24 h and 48 h. Cell viability was determined using a CCK-8 assay (APExBIO, USA). For live/dead cell staining, the cells were processed as previously described.

**RNA sequencing (RNA-seq)**

The RNA extraction process involved utilizing the TRIzol reagent (Thermo Fisher Scientific Inc.). Subsequently, preparation of the RNA-seq libraries was conducted using the KAPA Stranded RNA-Seq Library Prep Kit (Illumina, San Diego, CA, USA). The sequencing operation was conducted on the Illumina NovaSeq^TM^ 6000 platform, facilitated by LC Bio-Technology CO., Ltd (Hangzhou, China). Transcripts were identified where the expression levels exhibited a | log^2^ (fold change) | > 2 and an adjusted P-value < 0.05.

### Transmission electron microscopy (TEM)

Cells were prepared for TEM examination by fixation in a specialized solution designed for electron microscopy for 2-4 h. Then, the cells were embedded 1% agarose, subjected to dehydration, and cut into ultrathin sections with a 60-80 nm thickness using an ultramicrotome. These sections were stained for 15 min with a solution of 2% uranyl acetate saturated in ethanol and lead citrate and left to dry at room temperature overnight. Imaging was conducted using a TEM (Hitachi-7800 system).

### Western blot analysis

Cells of the 4T1 line were cultured in 6-well plates at a density of 1 × 10^4^ cells per well and treated accordingly. Following the predetermined time intervals, cellular proteins were harvested, and their concentrations were determined by employing a BCA protein assay kit (Beyotime, China). Equivalent quantities of the proteins were resolved by SDS‒PAGE and electro-transferred onto PVDF membranes (Millipore, USA). Antibodies specific to the proteins of interest, including AMPK, p-AMPK, Beclin1, p-Beclin1, SLC7A11, tubulin, GAPDH (Proteintech), and GPX4 (Abmart), were utilized for detection. Protein bands were made visible through a chemiluminescence detection system, and the intensity of the bands was quantified with ImageJ software.

### Co-immunoprecipitation (Co-IP)

The cellular extracts were prepared by lysing the cells in a buffer enriched with protease inhibitors on ice, followed by centrifugation to remove cellular debris. The lysates were kept at 4 °C overnight for incubation with either IgG or specific antibodies. Magnetic beads (BEAVER) were then introduced and allowed to bind for 2 h at 4 °C. The immunoprecipitates were rinsed with lysis buffer, and the bound proteins were released by boiling in a sample buffer. The extracted proteins were analyzed using Western blot.

### Molecular docking analysis

The crystal structures of BECN1 (PDB ID: AF-O88597-F1) and SLC7A11 (PDB ID: AF-Q9WTR6-F1-v4) were obtained from the AlphaFold database. Docking sites were identified through the EMBL-EBI platform, and the docking results were visualized using PyMOL software.

### Glutathione (GSH) assay

The levels of intracellular GSH were quantified with the aid of a GSH assay kit supplied by Sigma-Aldrich. The cells were treated with a 5% solution of 5-sulfosalicylic acid after a wash with cold PBS. The lysed cells were centrifuged at 10,000 g for 10 min, and the supernatant was collected. Then, the mixture was supplemented with 150 μL of the GSH working solution and incubated for 5 min at room temperature. After this time, 50 μL of diluted solution containing nicotinamide adenine dinucleotide phosphate (NADPH) was introduced. The optical density of the formed yellow compound was then determined at 412 nm with the aid of a microplate reader (Thermo Fisher Scientific Inc.).

### GPX4 activity assay

To quantify the activity of GPX4, a Total Glutathione Peroxidase Assay Kit (Beyotime) was employed. The cells were first rinsed with cold PBS and lysed with a Cell Lysis Buffer (Beyotime), and samples were subjected to centrifugation at 12,000 g for 10 min at 4 °C. Then, the supernatant was harvested for a BCA protein quantification assay. 40 μL of GPX4 assay working solution and GPX4 Assay Buffer 50 μL were added to each sample and incubated to facilitate the reduction of GSSG at room temperature for 15 min. Subsequently, 10 μL of 30 mM peroxide reagent was introduced to each sample. Immediately measure the absorbance at 340 nm kinetically every 1 min for 5 min via a microplate reader supplied by Thermo Fisher Scientific Inc.). GPX4 activity was calculated using the appropriate formula provided in the kit instructions.

### Lipid ROS assay

The measurement of lipid ROS levels was conducted using the C11-BODIPY probe from Dojindo, adhering to the provided protocol. The cells were treated with 1 mL of the C11-BODIPY solution for 60 min at a temperature of 37 °C, followed by two washes with PBS. After resuspending the cells in 500 μL of PBS, the lipid ROS levels were assessed using a fluorescent cell imager and flow cytometry (FCM).

### *In vivo* experiment

On the first day of the experiment, mice were randomly divided into 5 groups (n = 5 per group): (1) Control, (2) IMSFs, (3) IMSFs+TAT, (4) F@SF-HA+AMF, (5) IMSFs+AMF, and (6) IMSFs+AMF+TAT. Tumors in the designated groups were injected with F@SF-HA or IMSFs, followed by AMF exposure the following day. TAT-Beclin1 was injected once daily for two consecutive days, followed by a three-day interval. This cycle was repeated five times. Tumor volumes and body weights of the tumor-bearing mice were monitored every 3 d throughout the treatment cycle. Tumor volume was calculated as previously described. The mice underwent survival analysis and were observed until day 40. Mice were euthanized when tumor volumes reached 1500 mm³. Lung tissues were collected post-mortem, fixed, and stained with Bouin's solution to evaluate lung metastasis in each group.

### *In vivo* analysis of immune activation

Samples from the lymph nodes and tumors of mice were utilized to assess the levels of immune activation. These samples were finely chopped and transferred into a centrifuge tube containing PBS (pH 7.4) supplemented with 2% fetal bovine serum. A single-cell suspension was created using a homogenizer that applied mild pressure, and this process did not involve using digestive enzymes. Lysis buffer for red blood cells (RBCs) was employed to remove RBCs, after which the residual cells were labeled with fluorescent antibodies. For the evaluation of the dendritic cells (DCs) maturation, the prepared single-cell suspension was stained with FITC-conjugated anti-CD11C, APC-conjugated anti-CD86, and PE-conjugated anti-CD80, following the standard staining protocol. Effector memory T cells (TEM cells) were analyzed by immunostaining the suspension with APC-conjugated anti-CD3, PE-conjugated anti-CD8, and FITC-conjugated anti-CD4.

### Cytokine detection

Samples of the supernatants were taken from both the cultures of DC cells and the mic sera. TNF-α, IFN-γ, IL-12, and IL-6 concentrations within these samples were measured using their respective ELISA kits (ELK Biotechnology), adhering to the guidelines outlined in the manufacturer's protocols.

### *In vivo* synergistic therapy of tumors

After completing the treatment, tumors were collected from the mice. The primary tumors were homogenized into a fine powder using liquid nitrogen, and protein was isolated from the tissue. Western blotting was performed to assess the expression levels of P62 and LC3II in the tumor samples. Tumor tissues were subjected to histological analysis, including H&E staining, Ki-67 antibody, TUNEL assay, GPX4 antibody, and SLC7A11 antibody staining. Additionally, immunohistochemical staining for CD8^+^ T cells was performed to assess the immune response in the tumor's microenvironment. Immunofluorescence intensity was quantified using ImageJ software.

### *In vivo* biosafety

6-week-old BALB/c mice in good health were allocated randomly into 5 distinct groups (n = 3 per group). One group remained the control, without any injection, while the other four received IMSFs and TAT-Beclin1 injections. Serum and blood were obtained on days 1, 7, 14, 21and 28 for hematological and biochemical evaluations. These analyses were conducted using an automatic biochemical analyzer (Chemray 240) and an automatic hematology analyzer (BC-2800 VET). The major organs, including the lungs, heart, liver, spleen, and kidneys, were harvested and stained with H&E for histological evaluation.

### Statistical analysis

Utilizing GraphPad Prism 10 software, the data collected from this investigation were subjected to analysis. Results are expressed as mean values accompanied by standard deviations (SDs). Both one-way ANOVA and Student's t-test were utilized to compare different groups. Statistical significance was indicated as follows: *p < 0.05, **p < 0.01, ***p < 0.001, and #p < 0.05, ##p < 0.01, ###p < 0.001.

## Supplementary Material

Supplementary figures.

## Figures and Tables

**Table 1:** Composition of F@SF-HA and IMSFs hydrogels

Groups	Powder (mg)		SF-HA hydrogels (mL)
	Fe_3_O_4_	Sorafenib	
F@SF-HA	64.1	0	1
IMSFs-4%Fe3O4	42.1	10.53	1
IMSFs-6%Fe3O4	64.1	10.75	1
IMSFs-8%Fe3O4	87.1	10.99	1

## Abbreviations

TNBC: triple-negative breast cancer
SF: silk fibroin
IMSFs: injectable magnetic thermoresponsive silk fibroin-based hydrogels
TEM: transmission electron microscopy
AMF: alternating magnetic field
ROS: reactive oxygen species
GPX4: glutathione peroxidase 4
ICD: immunogenic cell death
IFN-γ: interferon-gamma
LPO: lipid peroxidation
PUFAs: polyunsaturated fatty acids
GSH: glutathione
SOR: sorafenib
HCC: hepatocellular carcinoma
EPR: enhanced permeability and retention
HA: hyaluronic acid
Fe₃O₄: superparamagnetic iron oxide nanoparticles
SF-HA: silk fibroin-hyaluronic acid
MHT: magnetic hyperthermia temperature
MTT: mild magnetic hyperthermia therapy
ALT: alanine aminotransferase
AST: aspartate aminotransferase
ALB: albumin
H&E: hematoxylin and eosin
BDDE: 1,4-butanediol diglycidyl ether
LiBr: lithium bromide
SEM: scanning electron microscopy
EDS: energy dispersive spectroscopy
XRD: X-ray diffraction
FTIR: fourier-transform infrared
XPS: X-ray photoelectron spectroscopy
ICP-MS: inductively coupled plasma mass spectrometry
TGA: thermogravimetric analysis
PBS: phosphate-buffered saline
Co-IP: co-immunoprecipitation
DAMPs: damage-associated molecular patterns
HMGB1: high-mobility group box 1
CRT: calreticulin
APCs: antigen-presenting cells
TLR4: toll-like receptor 4
DCs: dendritic cells
CTL: cytotoxic T lymphocyte
LNs: lymph nodes
