# Supplementary material for: Injectable SF-platform orchestrates GPX4-targeted ferroptosis-autophagy-immunogenic circuit for overcoming oxidative resistance in triple-negative breast cancer
Source: Theranostics. 2025 Aug 11;15(17):8757–78. doi: 10.7150/thno.116013 (PMC12439161; doi:10.7150/thno.116013)
Supplement: Supplementary file 1 — Supplementary figures. [file thnov15p8757s1.pdf]

Injectable SF-platform orchestrates GPX4-targeted ferroptosis-autophagy-immunogenic circuit for overcoming oxidative resistance in triple-negative breast cancer

Hui Yuan [a, b, 1], Xiongwu Li [c, 1], Muhua Yu [d], Youde Cao [a, b], Lingcheng Wu [a], Suyujie Shi [a], Zuying Li [a], Yaying Yang [a, b *], Kexiao Yu [e, **], Bing Liang [a, b, ***]

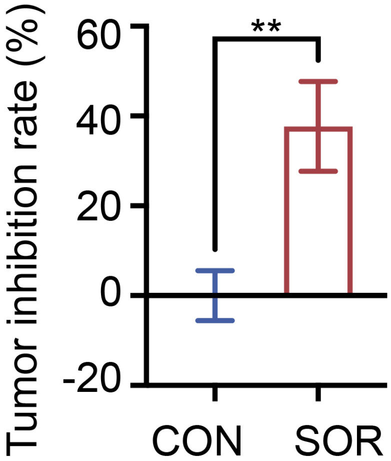

Figure S1. Tumor inhibition rate of 4T1 tumor-bearing mice with the saline and sorafenib treatments groups. (n = 3 per group, **p < 0.01 in comparison between CON and SOR).

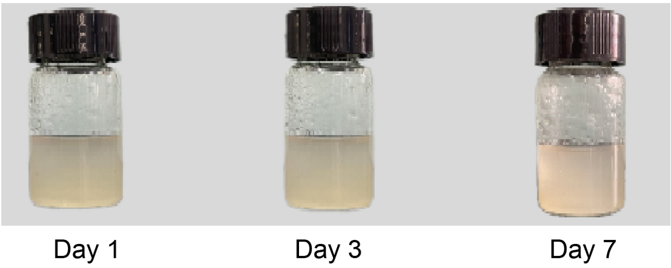

Figure S2. The pictures of SF-HA hydrogel standing for 1, 3 and 7 days.

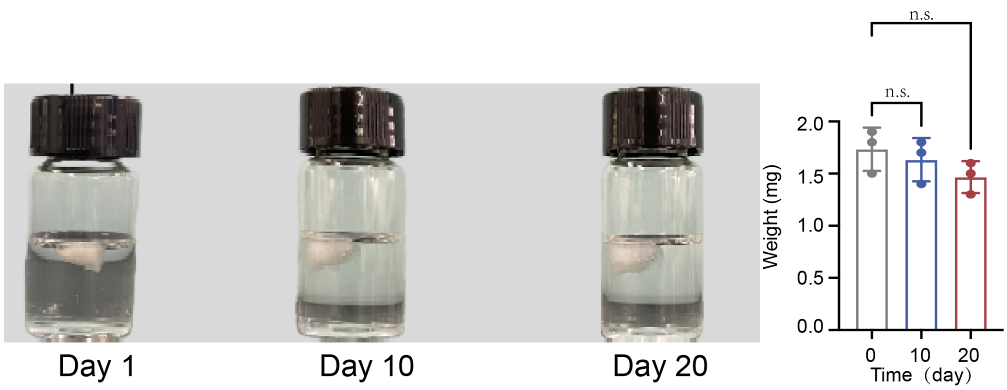

Figure S3. Pictures of placed SF-HA hydrogel freeze-dried in PBS and the weight of the freeze-dried on the 1st, 10th and 20th day. (The data are shown as the means ± SDs, n = 3 per group, n.s. represented no significance)

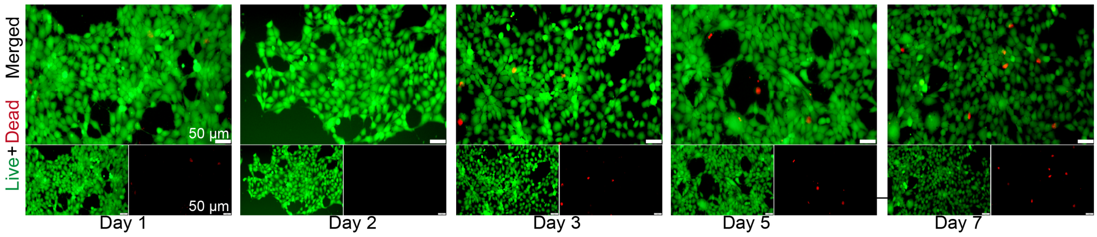

Figure S4. Live (green)/dead (red) fluorescence images of 4T1 cells cultured with SF-HA hydrogel for 1, 2, 3, 5 and 7 days.

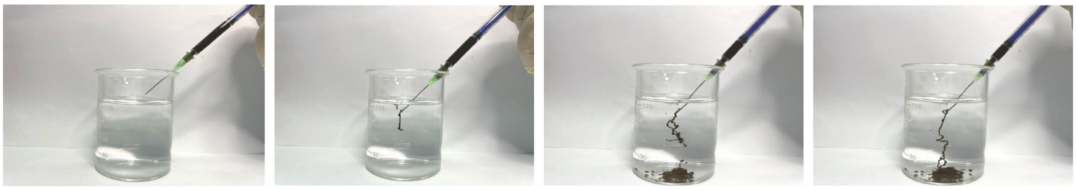

Figure S5. The images of IMSFs hydrogel freely passes through the 1 mL syringe.

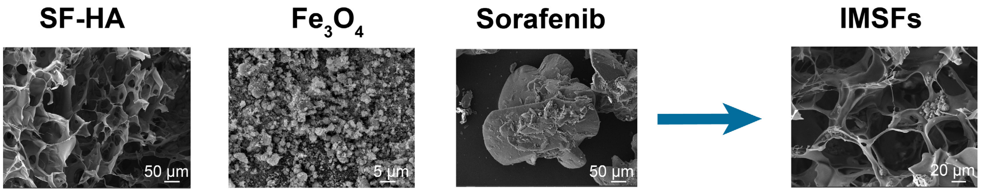

Figure S6. SEM images of SH-HA, Fe_3O_4, sorafenib and IMSFs.

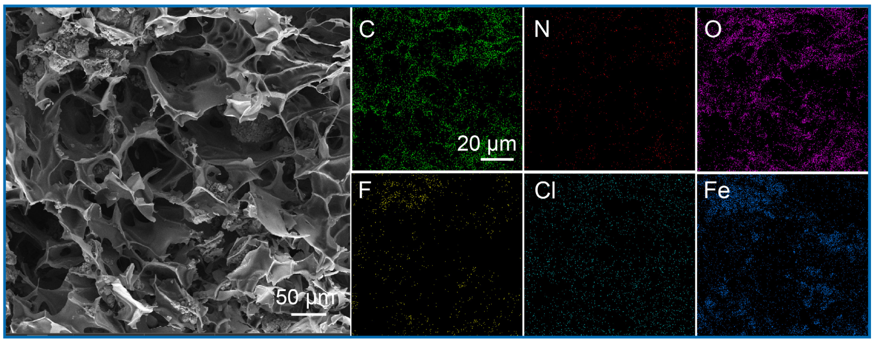

Figure S7. SEM images and corresponding mapping images of IMSFs without AMF exposure.

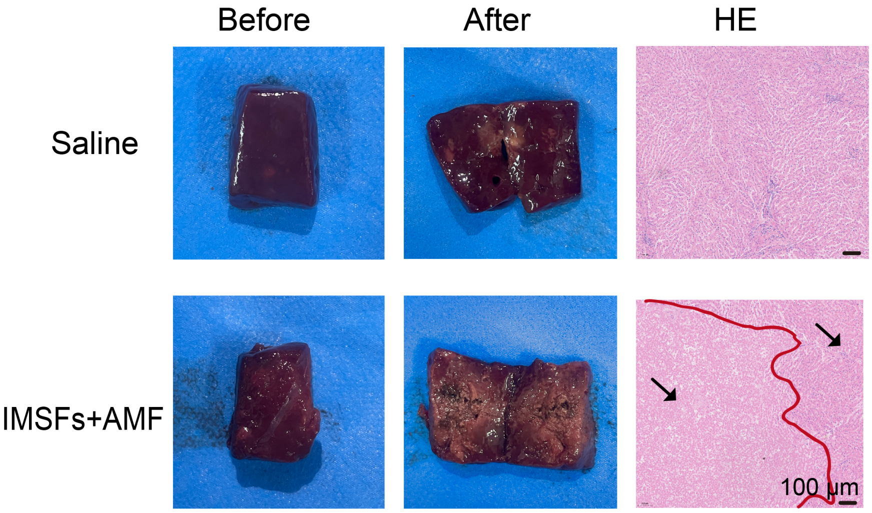

Figure S8. In vitro bovine liver digital images and H&E before and after magnetic field exposure between saline and IMSFs groups.

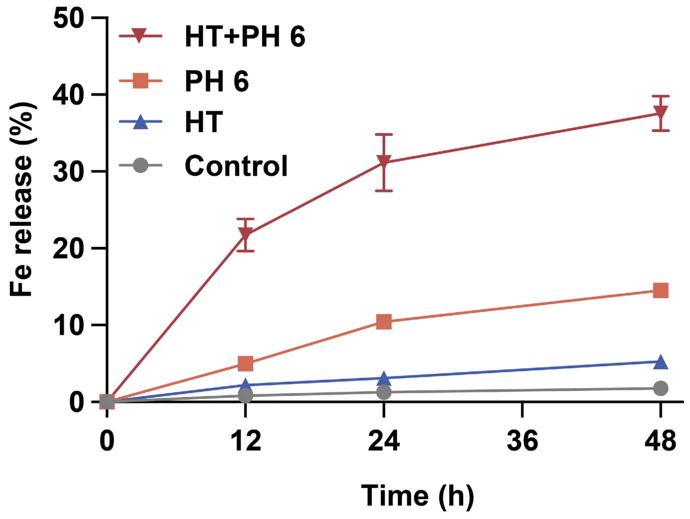

Figure S9. Cumulative release of Fe from IMSFs at different groups.

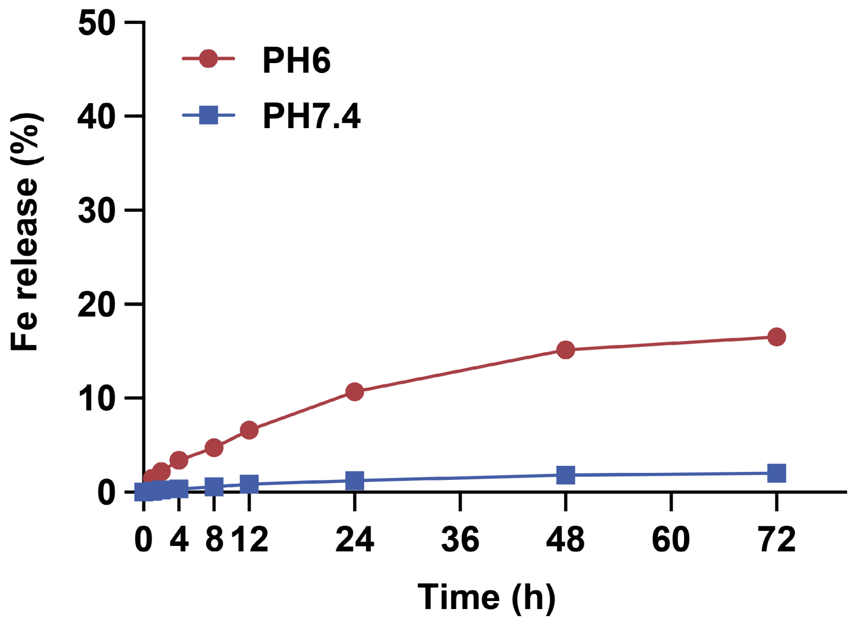

Figure S10. Cumulative release of Fe from IMSFs at different pH without AMF exposure.

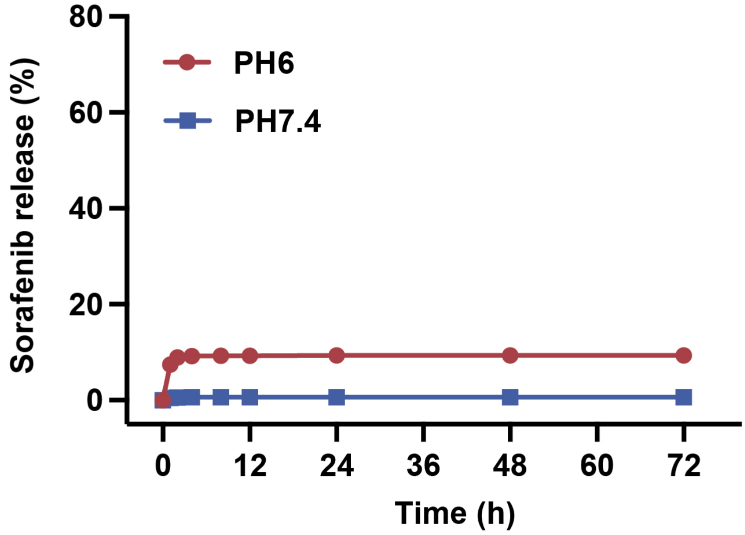

Figure S11. Cumulative release of sorafenib from IMSFs at different pH without AMF exposure.

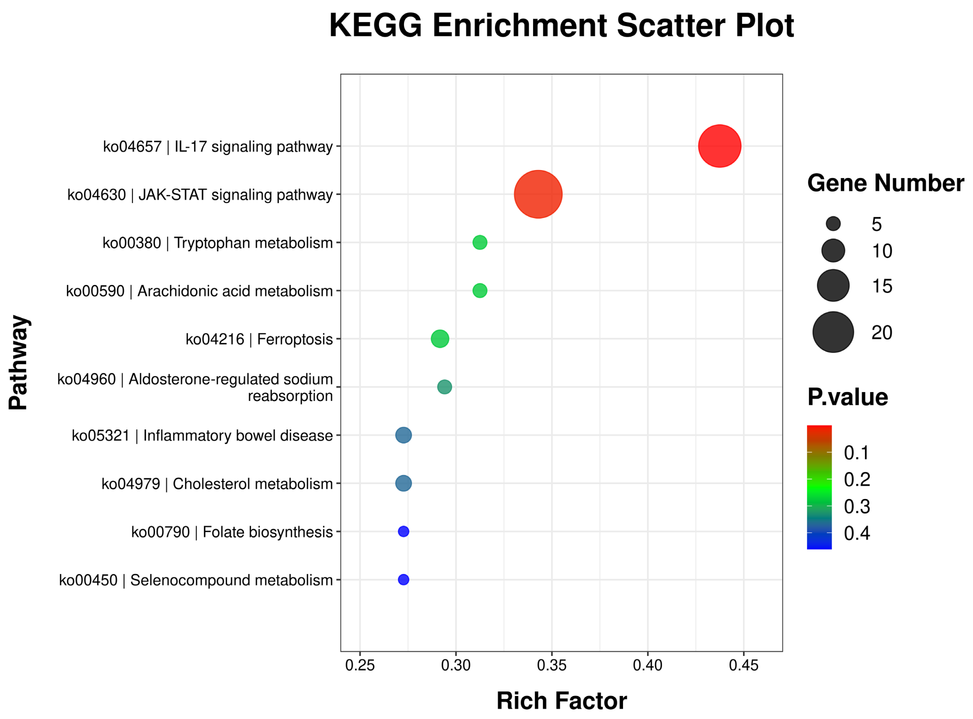

Figure S12. Representative KEGG pathways associated with genes that were significantly differentially expressed between the control and IMSFs+AMF groups.

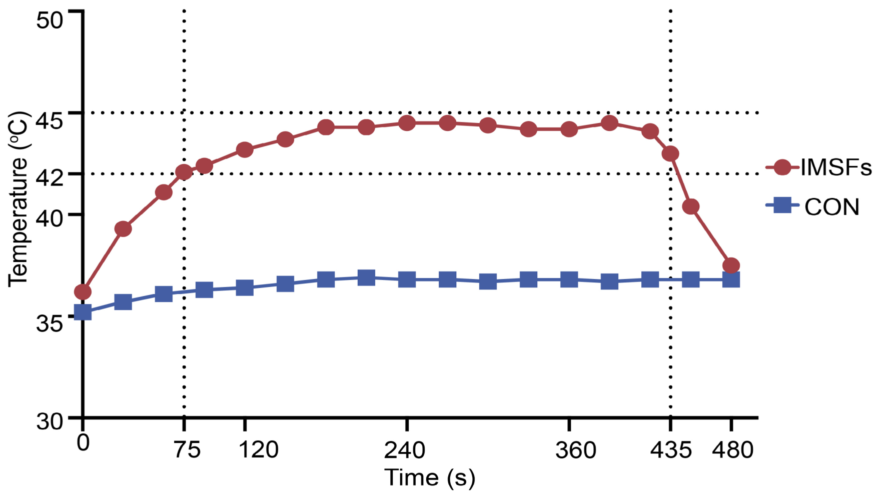

Figure S13. Corresponding quantitative temperature curves of 4T1 tumor-bearing mice after intratumoral injection of saline and IMSFs under AMF exposure.

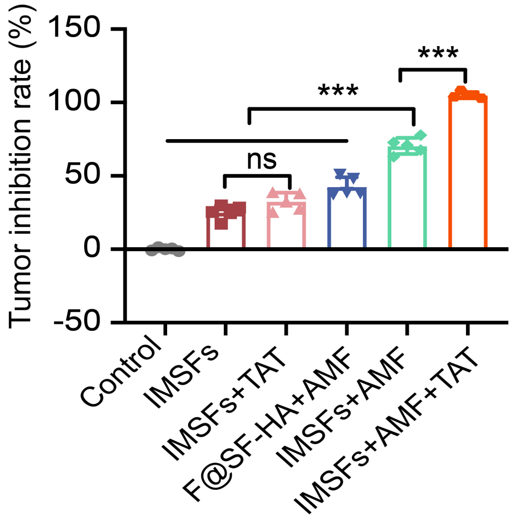

Figure S14. Tumor inhibition rate of 4T1 tumor-bearing mice with the various treatments. (The data are shown as the means ± SDs, n = 5 per group, n.s. represented no significance, ***p < 0.001 in comparison with the IMSFs+AMF groups, respectively.)

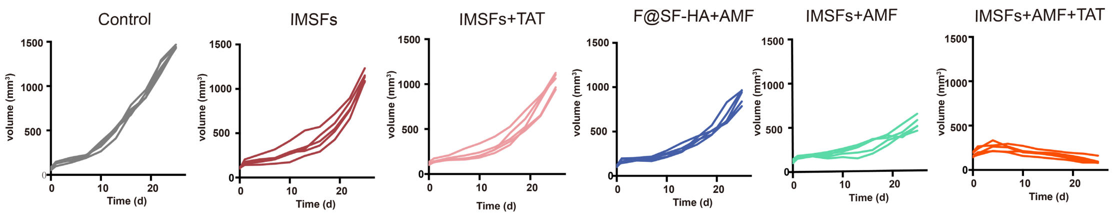

Figure S15. Primary tumor growth curves of mice in each group.

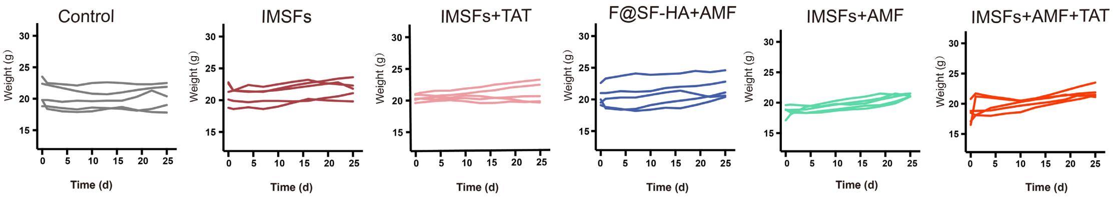

Figure S16. Body weight change curves of mice in each group.

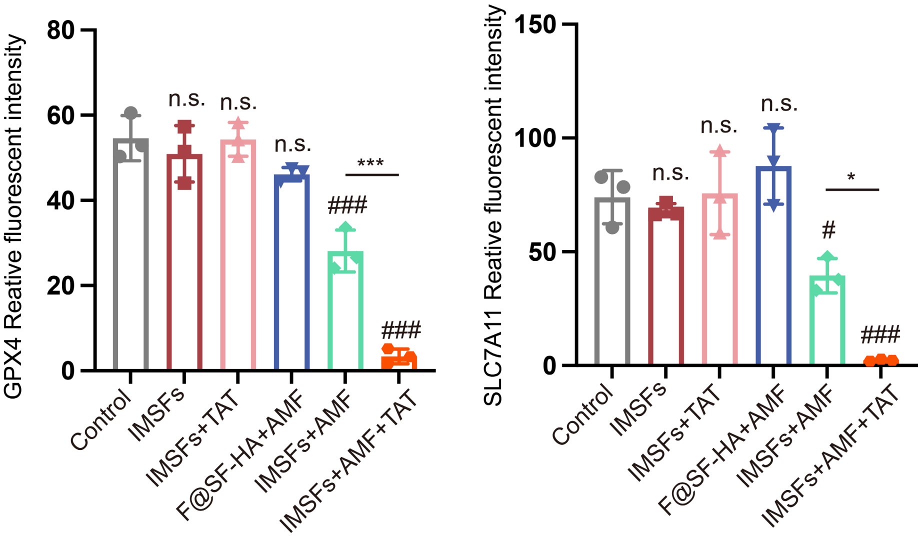

Figure S17. Analysis of the representative fluorescence intensities of GPX4 and SLC7A11 in tumor tissue sections from each group after different treatments. (The data are shown as the means ± SDs, n = 3 per group, n.s. represented no significance, #p < 0.05, ###p < 0.001 when compared to the control groups, and *p < 0.05, ***p < 0.001 for the distinction between IMSFs+AMF and IMSFs+AMF+TAT groups.)

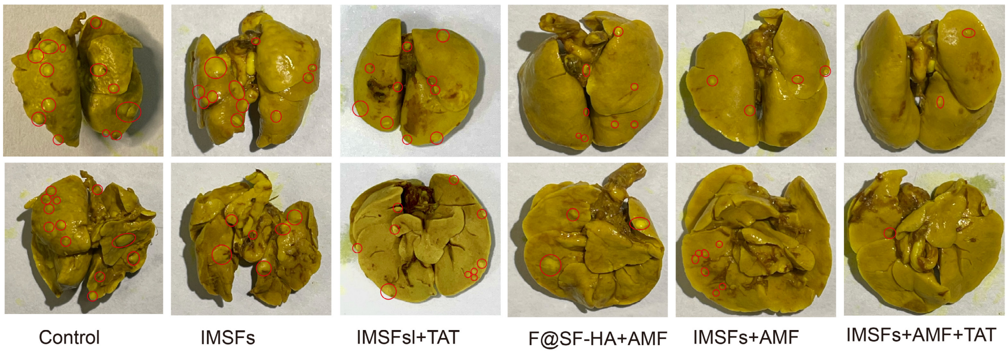

Figure S18. Digital photographs staining images of representative pulmonary metastatic nodules (circles) from the mice in each group at the end of treatment.

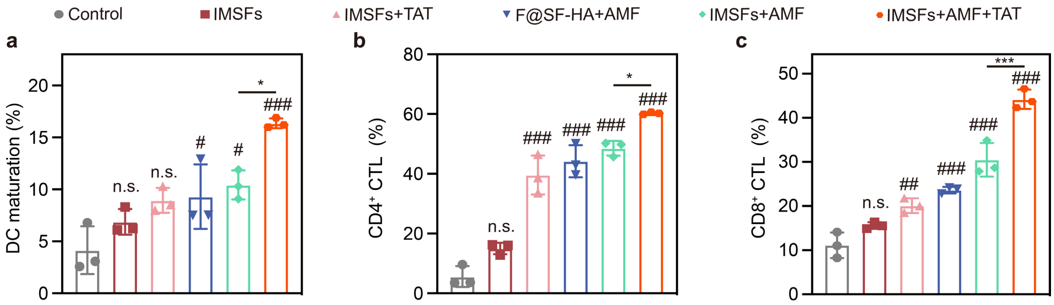

Figure S19. FCM quantification analysis of DC maturation and CTLs in drain LNs adjacent to the primary tumors. (The data are shown as the means ± SDs, n = 3 per group, n.s. represented no significance, #p < 0.05, ##p < 0.01, ###p < 0.001 when compared to the control groups, and *p < 0.05, ***p < 0.001 for the distinction between IMSFs+AMF and IMSFs+AMF+TAT groups.)

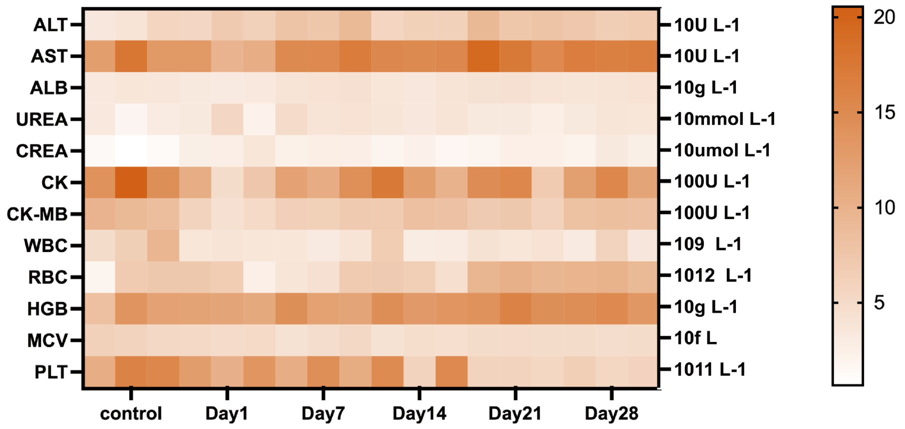

Figure S20.Heat map of blood routine and blood biochemical indexes after different treatment in 0,1,7,14,21and 28 days.

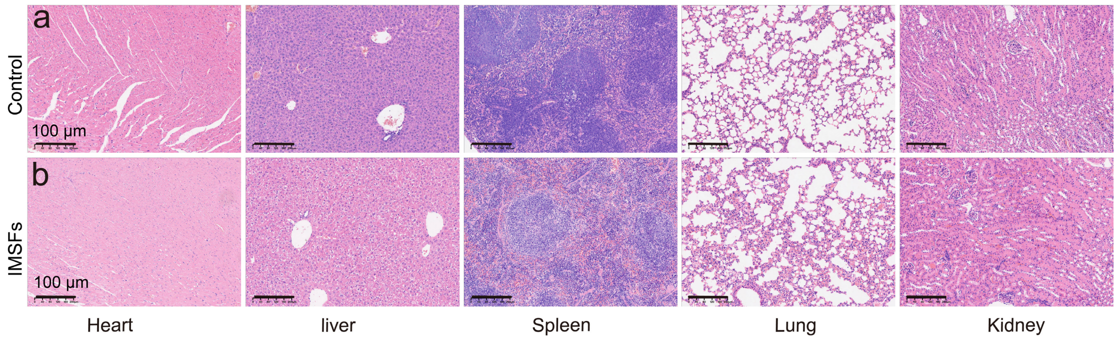

Figure S21. Representative H&E images of major organs (heart, liver, spleen, lung and kidney) of BALB/c mice between Control and IMSFs groups on 28th.
